# Human iPSC-derived renal organoids engineered to report oxidative stress can predict drug-induced toxicity

**DOI:** 10.1016/j.isci.2022.103884

**Published:** 2022-02-07

**Authors:** M.L. Lawrence, M. Elhendawi, M. Morlock, W. Liu, S. Liu, A. Palakkan, L.F. Seidl, P. Hohenstein, A.K. Sjögren, J.A. Davies

**Affiliations:** 1 Deanery of Biomedical Sciences, University of Edinburgh, Hugh Robson Building, George Square, Edinburgh, EH8 9XD UK; 2 Clinical Pathology Department, Faculty of Medicine, Mansoura University, Mansoura, Egypt; 3 R&D Graduate, R&D, AstraZeneca, Gothenburg, Sweden; 4 SynthSys Centre for Synthetic and Systems Biology, UK Centre for Mammalian Synthetic Biology, School of Biological Sciences, University of Edinburgh, C.H Waddington Building, Max Born Crescent, Edinburgh, EH9 3BF, UK; 5 Leiden University Medical Center, Leiden University, Leiden, the Netherlands; 6 The Roslin Institute, The University of Edinburgh, Midlothian, UK; 7 CVRM Safety, Clinical Pharmacology and Safety Science, R&D, AstraZeneca, Gothenburg, Sweden

**Keywords:** Molecular medicine, Cellular toxicology, Cell biology

## Abstract

Advances in regenerative medicine have led to the construction of many types of organoids, which reproduce important aspects of endogenous organs but may be limited or disorganized in nature. While their usefulness for restoring function remains unclear, they have undoubted usefulness in research, diagnostics, and toxicology. In toxicology, there is an urgent need for better models for human kidneys. We used human iPS-cell (hiPSC)-derived renal organoids to identify HMOX1 as a useful marker of toxic stress via the oxidative stress pathway, and then constructed an HMOX1 reporter in hiPSCs. We used two forms of hiPSC-derived HMOX1-reporter renal organoids to probe their ability to detect nephrotoxicants in a panel of blind-coded compounds. Our results highlight the potential usefulness, and some limitations, of HMOX1-reporter renal organoids as screening tools. The results may guide development of similar stress-reporting organoid assays for other stem-cell-derived organs and tissues.

## Highlights

• Nephrotoxicants (gentamicin and cisplatin) make kidney organoids upregulate HMOX1
• We made a HMOX1 reporting human iPSC line (red fluorescent)
• We made kidney organoids from this reporter line
• The organoids detected toxicants in a blind-coded panel

## Graphical abstract

**Figure:**
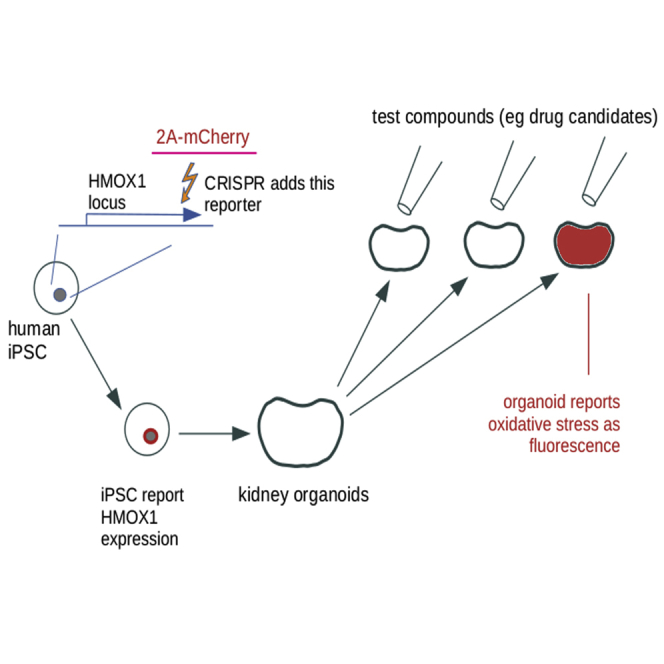

## Introduction

Nephrotoxicity is a challenging problem in translating drug development to clinical use. A significant proportion of acute and chronic kidney disease is caused by therapeutics in current clinical use. Off-target effects of prescribed drugs cause 19%–25% of kidney injury in critically ill hospital patients (Mehta et al., 2004; Uchino et al., 2005). A study at the turn of this century found 1% of all hospital patients over the age of 60 to have suffered drug-induced renal injury (Kohli et al., 2000). The nephrotoxicity of many common drugs arises from the inherent vulnerability of kidney compartments (especially proximal tubules) to toxic insult, caused mainly by the transport systems of these cells concentrating drugs in their cytoplasm. The poor predictive power of animal tests used during drug development, coupled with a lack of established *in vitro*, human-based screens, contributes to difficulties in identifying drug toxicity. Meta-analyses have suggested that correct predictions are made only 50% of the time using animal studies (Fletcher, 1978; Knight, 2008; Hartung, 2009; Archibald et al., 2011). Poor prediction by animal experiments, in part due to inter-species differences in renal transporter expression (Chu et al., 2013; Zou et al., 2018), results in withdrawal of many drugs after their expensive development. It may also explain the high nephrotoxicity of many licensed medicines.

Human cell cultures provide useful test-beds but they have limitations. Primary cultures from human kidneys (for example, biopsies, or organs harvested for transplant but not used) are useful but suffer from problems of reliable supply (Li et al., 2003; Jenkinson et al., 2012; Baverel et al., 2013; Jang et al., 2013; Weber et al., 2016). Recently, developments in stem cell technology have suggested an alternative approach of testing candidate compounds on renal tissue made from human stem cells. Several laboratories have developed methods to differentiate mouse ES cells and human-induced pluripotent cells (hiPSCs) to renal lineages and renal organoids (Takasato et al., 2015; Morizane et al., 2015; Kim and Dressler, 2005). Testing the response of these organoids to nephrotoxicants has provided pilot data suggesting renal organoids have potential as an assay system (Xinaris et al., 2015; Huch et al., 2017; Davies and Lawrence, 2018). Organoids are multicellular entities with many cell types and realistic micro-anatomy, and may therefore be more physiologically relevant than simple 2D monocultures. Their main disadvantages are variability between organoids and a labor-intensive production process, though recent efforts have been made to make small organoids in a semi-automated way (Przepiorski et al., 2018). From the point of view of toxicity testing, the anatomical complexity of organoids can be a drawback, because it makes obtaining a simple quantitative response to toxic stimuli challenging.

In this report, we address the latter problem by designing and constructing a fluorescent reporter system in hiPSCs that reports nephrotoxicity. We first used an established protocol (Takasato et al., 2015) to produce hiPSC-derived human renal organoids and exploited them to identify genes induced by a known test toxicant using RNA-seq. Of the genes identified, *HMOX1* (also known as *H O -1*, a key component of the oxidative stress response) seemed the most promising reporter of stress, and we therefore constructed a fluorescent reporter system to monitor activity of this promoter in hiPSCs. Oxidative stress (OS) can be a direct cause of nephrotoxicity or a secondary effect of toxicity. Thus, this reporter is designed to identify a broad and prominent class of nephrotoxicant, in which the OS response is activated, though it cannot be said to be exhaustive for identification of renal toxicity. Renal organoids were constructed from these genetically modified hiPSCs and their ability to detect nephrotoxicants correctly in a blind-coded panel of compounds was tested.

## Results

### Transporter expression in renal organoids and identification of HMOX1 as a potential reporter of toxic insult

**Figure 1:**
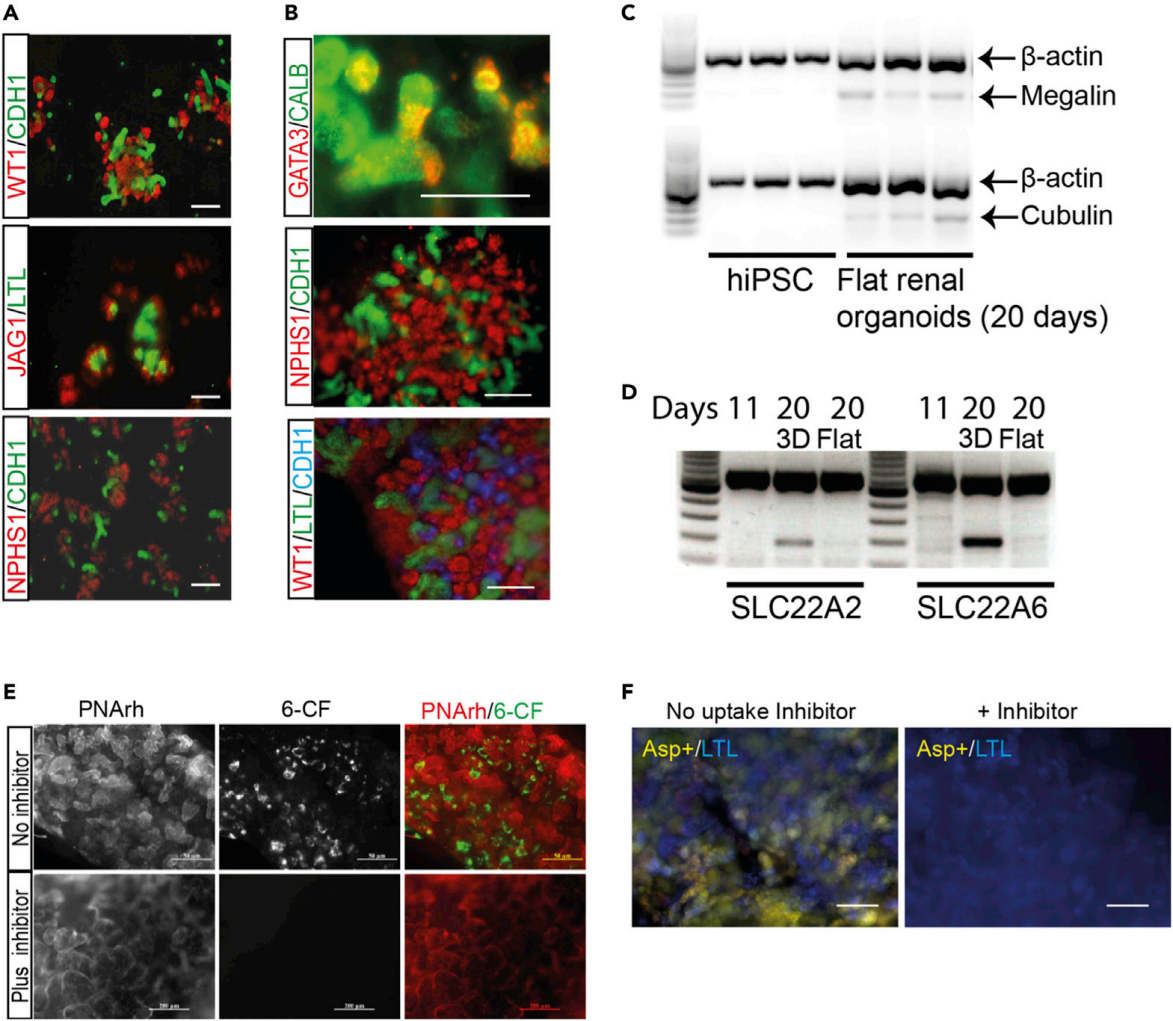
Renal organoids express renal markers and display some renal functions (A) Flat renal organoids express the renal mesenchymal and collecting duct markers WT1 and CDH1, podocyte marker NPHS1, and the nephron marker JAG1, as well as binding the proximal tubule (PT) marker lotus tetragonolobus lectin (LTL). (B) 3D renal organoids express markers of ureteric-bud tips (GATA3), collecting duct (CALB and CDH1), podocytes (WT1, NPHS1), and bind the PT marker LTL. (C) In contrast to undifferentiated hiPSCs, flat renal organoids express *Megalin* and *Cubulin* as measured by RT-PCR with three biological replicates each (3 different hiPSC samples and three flat organoids). (D) Expression of the renal anion and cation uptake transporters *OCT2 (SLC22A2)* and *OAT1 (SLC22A6)* in flat and 3D renal organoids by RT-PCR. (E) Specific tubular uptake of the fluorescent anion 6-CF (green) in 3D renal organoids stained with the live tubular marker rhodamine-peanut agglutinin (PNArh, red) either with or without the anion transporter inhibitor probenecid. (F) Specific tubular uptake of the fluorescent cation 4-(4-dimethylamino-styryl)-N-methylpyridinium iodide (Asp+, yellow) stained with the renal tubular marker lotus tetragonolobus lectin (LTL, blue) either with or without the cation transporter inhibitor TPA. Scale bars are 100 μm (A); 200 μm (B, top panel) and (E); 50 μm (B, middle and bottom panel) and (F). Unlabeled tracks on RT-PCR gels are molecular weight markers

Renal organoids can be broadly cultured in two ways; as flat organoids, or as truly 3D organoids. Well-established protocols for doing this were used to produce our organoids (Figure S1) (Takasato et al., 2015, 2016). The flat renal organoids were differentiated from hiPSCs and grown in wells and, once differentiated, expressed WT1 (a marker of metanephric mesenchyme and podocytes (Armstrong et al., 1993; Kreidberg et al., 1993)) and CDH1 (an epithelial marker of ureteric bud and distal convoluted tubules (Lee et al., 2013)). They also expressed the glomerular podocyte marker, NPHS1, and the nephron tubule marker, JAG1. The organoids also bound the nephron tubule stain *Lotus tetragonolobus* lectin (LTL) (Hennigar et al., 1985; Barresi et al., 1988)) (Figure 1A). 3D renal organoids were made from a modified version of the flat organoid protocol by dissociating and pelleting flat organoids after 7 days of differentiation (Figure S1E). The 3D organoids expressed the podocyte marker NPHS1 and bound the tubule marker LTL. In addition, strongly WT1-positive structures indicated the presence of podocytes, and there was some evidence of CALB-positive structures indicating presence of ureteric-bud like tissue; this was supported by the co-expression of GATA3 and CALB in ureteric-bud tips (Figure 1B).

We tested the expression levels of some renal transporters in both the flat and 3D renal organoids. Flat and 3D organoids expressed *Megalin (LRP2)* and *Cubulin (CUBN)* (Figure 1C). Expression of *OCT2 (SLC22A2)* and *OAT1 (SLC22A6)*, basolateral transporters that are important for renal clearance of some xenobiotics, were also assessed by RT-PCR (Figure 1D). These transporters were more strongly expressed in the 3D organoids than the flat organoids. Transport activity for OCT2 and OAT1 in the 3D organoids was confirmed by performing inhibitable uptake assays (Lawrence et al., 2015; Çetinkaya et al., 2003) using the fluorescent anion 6-CF (Figure 1E) or the fluorescent cation Asp+ (Figure 1F). This demonstrates the presence of functional transporter proteins in the organoids and that the integrity of the tubular epithelia in healthy untreated organoids was not seriously compromised.

Although some transporters were more strongly expressed in the 3D organoids, the presence of *Megalin* and *Cubulin* in the flat organoids, combined with their increased suitability for high-throughput analyses, led us to adopt this form of organoid to identify a potential reporter for toxic insults in renal organoids. hiPSCs were differentiated into flat renal organoids, and their renal identity was verified by confirming expression of common renal markers (as described above and in the Methods section). We treated these flat renal organoids for 24 h with the known nephrotoxicant, gentamicin (Regec et al., 1986, 1989; El Mouedden et al., 2000), at 1 or 4 mg/mL, leaving some as vehicle-only controls (3 separate organoids per condition). Gentamicin was chosen based on the presence of *Megalin* and *Cubulin* in these organoids because they are the main endocytic receptors involved in gentamicin uptake into proximal tubule cells (Schmitz et al., 2002) and would be expected to be necessary for a realistic toxic response. The transcriptomes of renal organoids in these three conditions were compared using RNA-seq analyses.

Expression of *Megalin* and *Cubulin* was confirmed in the RNA-seq data using gene expression data obtained from the three control samples (Figure S2A). Our RNA-seq results (and other studies (Bajaj et al., 2018)) suggested that, while some important transporters were expressed as expected in flat organoids, others were barely present (Figure S2B). In agreement with our preliminary transporter RT-PCR results, *OAT1 (SLC22A6)* was not detected in the three untreated biological replicates tested. *OAT2 (SLC22A7)* and *OAT3 (SLC22A8)* were also not detected. The expression of *OCT2 (SLC22A2)*, the main transporter responsible for cation uptake on the basolateral membrane of proximal tubule cells, was very low in flat organoid samples. On the other hand, *OATP4C1 (SLC O 4C1)*, a basolateral organic transporter for anions over 350 kDa, that is expressed at higher levels in developing murine embryonic kidneys than in adult (Mikkaichi et al., 2004; Lawrence et al., 2015), was expressed more strongly (Figure S2B). *MRP2 (ABCC2)* and *MRP4 (ABCC4)*, which are the major apical transporters that mediate efflux of anions into the urine, were both expressed. *MATE1 (SLC47A1)*, *OCTN1*, *OCTN2 (SLC22A4* and *SLC22A5)*, and *P-gp (MDR1)*, the main cation transporters at the apical membrane, were also present (Figure S2C). Although there was little expression of *OCT2*, *MATE1*, an extrusion protein *in vivo*, has been shown to be capable of apical uptake of at least some cations *in vitro* (Tsuda et al., 2007).

**Figure 2:**
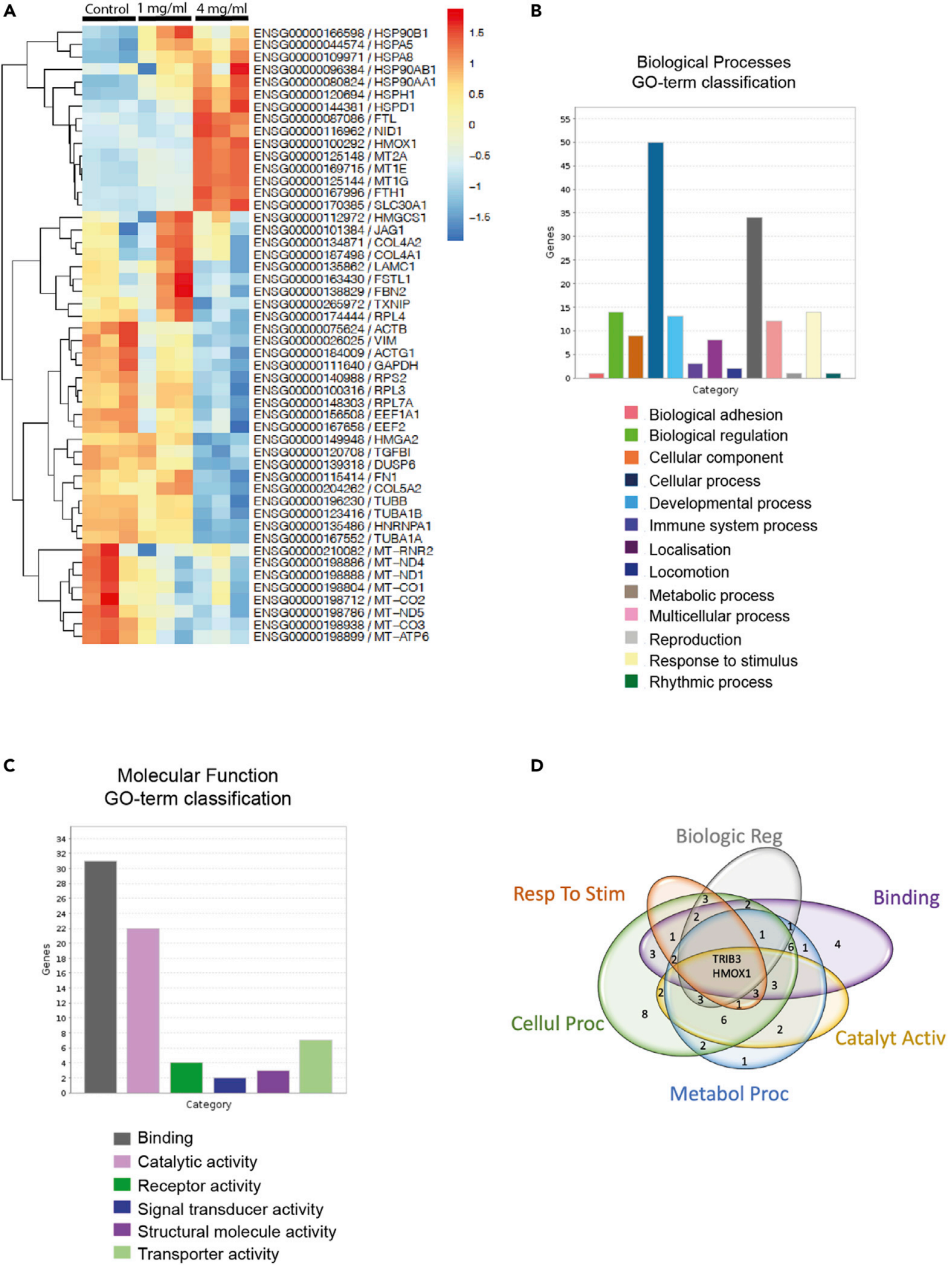
RNA-seq analyses of hiPSC-derived flat renal organoids (A) RNA-seq analyses of wild-type hiPSC-derived flat renal organoids treated with gentamicin (1 mg/mL or 4 mg/mL) or left untreated. Heatmap shows top 50 most differentially expressed genes among all three groups. The heatmap of each row is defined independently to span the highest and lowest normalized expression of that row (−1.5 (lowest) to 1.5 (highest)) thus showing the variability of that specific gene between the samples. (B and C) Gene ontology (GO) classification of the top 100 upregulated genes comparing the samples treated with the highest concentration with untreated samples (filtered first by lowest FDR and then top 100 genes with highest-fold increases) for biological processes or (C) molecular function. (D) The Venn diagram shows overlaps of genes within the six largest functional GO classifications

A heatmap of the most differentially expressed genes among all three groups (untreated, 1 mg/mL or 4 mg/mL) indicated that the profile of the 4 mg/mL gentamicin-treated samples was strikingly different from those of the other groups (Figure 2A). There were no genes expressed significantly differently between the control and the 1 mg/mL gentamicin-treated samples (applying a false discovery rate (FDR) cut-off of 0.05). In contrast, treatment with 4 mg/mL gentamicin caused the expression of 2,221 genes to increase and that of 1770 to decrease. Among the transcripts showing increased expression was *KIM-1* (log fold-change: 1.67; FDR-corrected p = 0.03), a biomarker commonly associated with renal tubular necrosis (Table S1). Expression of the oxidative stress marker *HMOX1* was also highly increased (log fold-change: 6.55; FDR-corrected p = 0.00015). Other transcripts related to apoptosis signaling and modulation were also highly upregulated including *LTA*, *JUN*, *FOS*, and *HRK*.

To further analyze the transcripts that had increased in the 4 mg/mL gentamicin-treated group compared to the control group, we used the 100 most-significantly increased transcripts to perform a gene ontology (GO-term) enrichment analysis using the PANTHER over-representation test (Mi et al., 2017). The GO terms of the enriched genes included classes highly relevant to toxic insults, including response to toxic substance, detoxification, regulation of cell death, cellular response to stress, response to unfolded and misfolded proteins, chaperone-mediated protein folding, and chaperone cofactor-dependent protein refolding (Table S2), implying that the renal organoids had been subjected to stress by the gentamicin exposure.

To find a potential marker that could be used as a reporter for toxicity, we performed a functional classification, using the PANTHER classification system (Mi et al., 2017). The functional classification was performed using the same 100 most-significantly increased transcripts in the high-concentration-treated group compared to the control group. The six most-represented gene ontology categories (Figures 2B and 2C) were identified. As functional groupings, these cannot be assigned p values, but the list of genes can also be analyzed using a statistical over-representation test using PANTHER, and these data, together with the p values, are provided in Table S2. The six ontology categories were further analyzed for the intersection of the genes between them to find a marker that could represent the most toxicity response mechanisms. Two genes were found to be common between all six groups: *TRIB3* (2.14 log-fold increase; FDR-corrected p = 2.15 × 10^−5^) and *HMOX1* (6.55 log fold-change; FDR-corrected p = 1.4 × 10^−4^) (Figure 2D and Table S5). Because *HMOX1* was found at the center of the functional classifications in our nephrotoxicant-treated renal organoids, and due to the weak evidence for more classical markers of nephrotoxicity, we decided to pursue *HMOX1* as a marker of toxicity in hiPSC-derived renal organoids.

We next treated renal organoids with increasing concentrations of the nephrotoxicant gentamicin, or cisplatin, to test the organoid expression of HMOX1 in response to known nephrotoxicant drugs. We also stained drug-treated organoids for HMOX1 together with LTL and COL IV (Figure S3A), or JAG1 (Figure S3B), to test whether the response is localized to a specific cell type in the organoid. HMOX1 expression increased in response to the toxic drugs but that increase was not limited to a specific renal structure. The general increase in HMOX1 expression in response to the toxic drugs, rather than specific response in proximal tubular cells, the primary site of toxicity of both drugs, could be due to the availability of the drug to all cell types of the organoid through the media in contrast to what happens *in vivo* where these drugs filter through the glomeruli then specifically accumulate inside the proximal tubular cells, through apical uptake pathways, where most of their toxic effect is seen.

### HMOX1 oxidative stress reporter hiPSC cell lines

**Figure 3:**
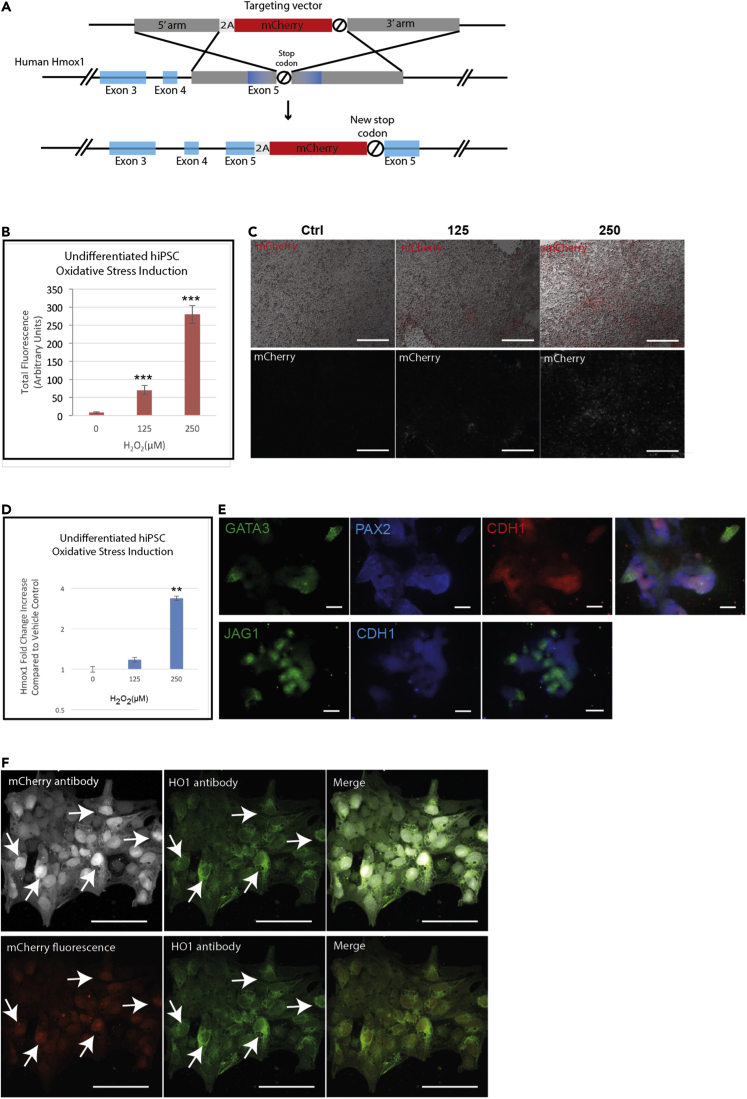
Construction and verification of *HMOX1* reporter hiPSCs (A) Targeting vector diagram showing insertion of 2A peptide-mCherry-STOP sequence replacing endogenous stop codon of human *HMOX1* in hiPSCs using CRISPR gene editing. Homologous arms upstream (5’: 500bp) and downstream (3’: 1049bp) of the stop codon are shown in gray (B and C) Increase in mCherry expression in undifferentiated *H O 1*-mCherry-hiPSCs after induction of oxidative stress with hydrogen peroxide by measuring total fluorescence and (C) representative images. Scale bars are 200 μm. (D) *HMOX1* transcript increase in undifferentiated *H O 1*-mCherry-hiPSCs after induction of oxidative stress (by qPCR). (E) *H O 1*-mCherry-hiPSC differentiated flat renal organoids express nephron markers (GATA3, PAX2, CHD1, and JAG1) and form renal structures. Scale bars are 100 μm. (F) Cellular co-expression of HO-1 and mCherry in undifferentiated hiPSCs after induction of oxidative stress with hydrogen peroxide (150 μM), either using anti-HMOX1 and anti-mCherry antibodies (top panel) or anti-HMOX1 antibody together with reporter mCherry fluorescence (bottom panel). Scale bars are 100 μM. In all graphs, data are represented as mean ± SEM, and p < 0.05 is indicated with (∗), p < 0.01 with (∗∗) and p < 0.001 with (∗∗∗)

Having identified *HMOX1* as an important marker of renal toxicity and confirmed its response to gentamicin in our renal organoids, we used CRISPR technology to insert a fluorescent reporter (mCherry) into the *HMOX1* locus to report on its expression. We included a 2A-peptide upstream of the mCherry sequence to uncouple the HMOX1 protein and the reporter protein (Figure 3A). Clonal lines were established and further experiments were carried out on a homozygously targeted clone (*H O 1*-mCherry-hiPSCs).

To test the response of the *H O 1*-mCherry-hiPSCs, still in their undifferentiated state, we treated one of the cloned hiPSC lines with different concentrations of hydrogen peroxide to induce oxidative stress (0, 125, or 250 μM) for 24 h. Fluorescence increased in a dose-dependent manner (Figures 3B and 3C). This increase in fluorescence reflected the increase in *HMOX1* transcripts in response to increasing hydrogen peroxide concentrations, measured by qPCR (Figure 3D). In order to verify that insertion of the reporter downstream of the *HMOX1* locus did not affect the ability of the cells to differentiate into flat renal organoids, we differentiated the *H O 1*-mCherry-hiPSC line into flat renal organoids, as we had for the hiPSC parent line. After 18–20 days of differentiation, differentiated cells were stained for the presence of renal markers (GATA3, JAG1, PAX2, and CDH1), confirming that the reporter hiPSCs could be differentiated into renal organoids (Figure 3E). To further validate the cellular co-expression of HMOX1 and mCherry, undifferentiated hiPSCs induced with 150 μM hydrogen peroxide were co-stained with either HMOX1 and mCherry antibodies (Figure 3F, top panel), or with HMOX1 antibody only and imaged together with mCherry fluorescence (Figure 3F, bottom panel).

### Renal organoids derived from the HO1-mCherry-hiPSC oxidative stress reporter cell line predict toxicity from known nephrotoxicants

The overall aim of this work was to generate renal organoids that would report toxic insults by means of an easily read fluorescent signal. To determine whether the reporter cell line could detect oxidative stress when the cells were differentiated into renal organoids, we treated them with gentamicin and cisplatin. These compounds are known to cause renal toxicity, either through direct induction of oxidative stress or as a secondary response to renal toxicity (Karatas et al., 2004; Miller et al., 2010; Morales et al., 2010). Importantly, these compounds gain entry to the cell through specific renal transporters, unlike the hydrogen peroxide used to induce oxidative stress in the undifferentiated hiPSCs. The concentrations of cisplatin and gentamicin chosen were based on previous *in vitro* renal toxicology studies (Regec et al., 1989; Jun et al., 2018; Bajaj et al., 2018).

**Figure 4:**
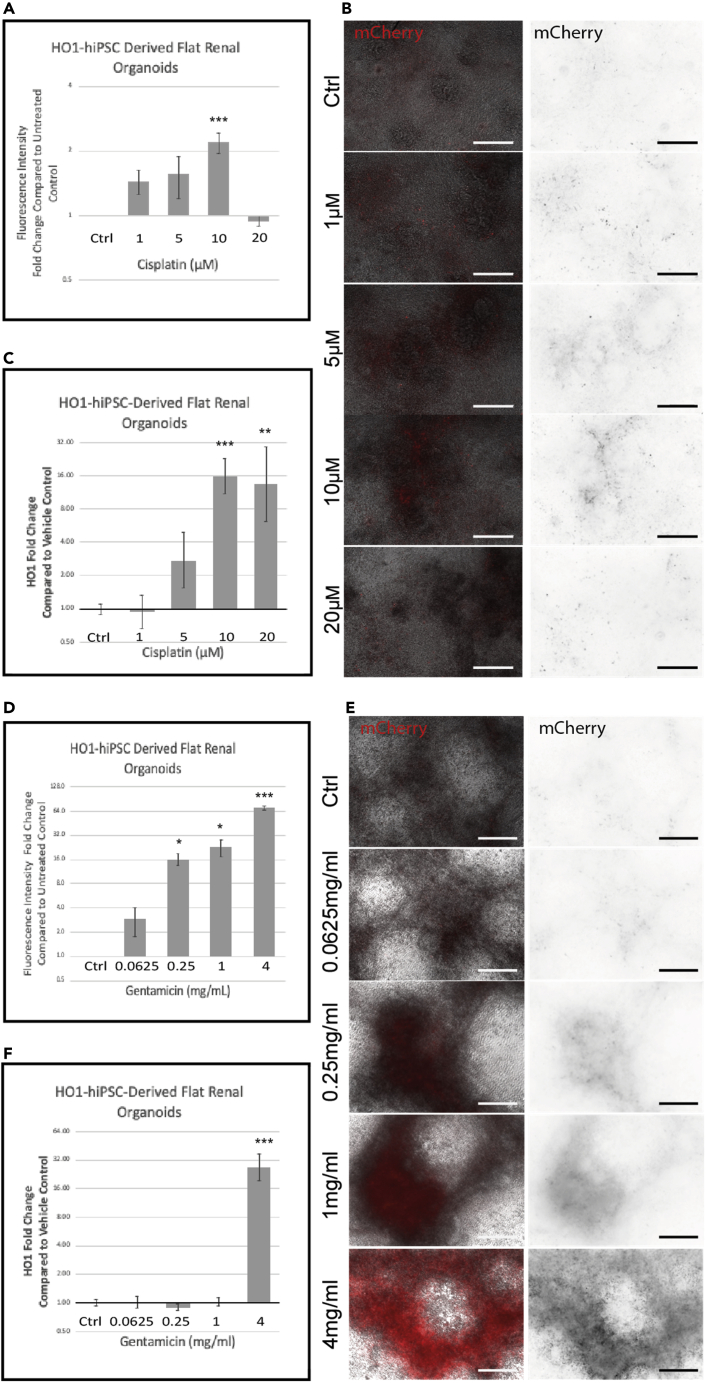
Response of *H O 1*-mCherry-hiPSC reporter-derived flat organoids to nephrotoxicants (A–F)Increase (fold-change relative to vehicle control) in mCherry expression in *H O 1*-mCherry-hiPSC-derived flat renal organoids treated with cisplatin (A) or gentamicin (D) and representative images for cisplatin (B) or gentamicin (E). Scale bars are 200 μm. *HMOX1* transcript increase (by qPCR) in *H O 1*-mCherry-hiPSC-derived flat renal organoids treated with cisplatin (C) or gentamicin (F). qPCRs are fold-change relative to vehicle control. Data are represented as mean ± SEM, p < 0.05 is indicated with (∗), p < 0.01 with (∗∗) and p < 0.001 with (∗∗∗)

Fluorescence intensity increased in a dose-dependent manner in the flat organoids treated with increasing amounts of either cisplatin (Figures 4A and 4B) or gentamicin (Figures 4D and 4E), with a similar increase in *HMOX1* gene expression measured by qPCR (Figures 4C and 4F). The increases in reporter and gene expression are not identical, probably because the half-lives of the mCherry fluorophore and the *HMOX1* transcript are not the same, with the fluorophore able to persist in the cell for longer (Shaner et al., 2004; Snapp, 2009). We also noticed that our cisplatin-treated organoids had a visibly greater loss of structure and tissue integrity than the gentamicin-treated organoids, probably due to the difference in mechanism of toxicity of the two compounds (data not shown).

### Response of HO1-mCherry-hiPSC-derived renal organoids to a blind-coded panel of drug compounds

Owing to the differential expression of various uptake and efflux transporters assessed by RT-PCR in both flat and 3D organoids, and by RNA-seq transcript analysis in flat organoids, we used both flat and 3D organoids to test the usefulness of the *H O 1*-mCherry-hiPSC line for predicting renal toxicity.

A panel of blind-coded compounds was produced, that included known nephrotoxic compounds, non-toxic compounds, and compounds that are not considered toxic to kidneys (but that might still be toxic to other organs). The compounds, selected and supplied by the Gothenburg authors without revealing their identities to the Edinburgh authors, were as follows: the control compound, not expected to be toxic, was dexamethasone. The example of a non-nephrotoxic compound that may be toxic to other organs was ketoprofen, which induces oxidative stress (Cheng et al., 2014) through effects on the mitochondria (van Leeuwen et al., 2012), and has its main toxic effect in the gastrointestinal tract. Examples of nephrotoxicants included puromycin, cisplatin, gentamicin, cidofovir, and ifosfamide (Ho et al., 2000; Barnett and Cummings, 2018; Akilesh et al., 2014).

**Figure 5:**
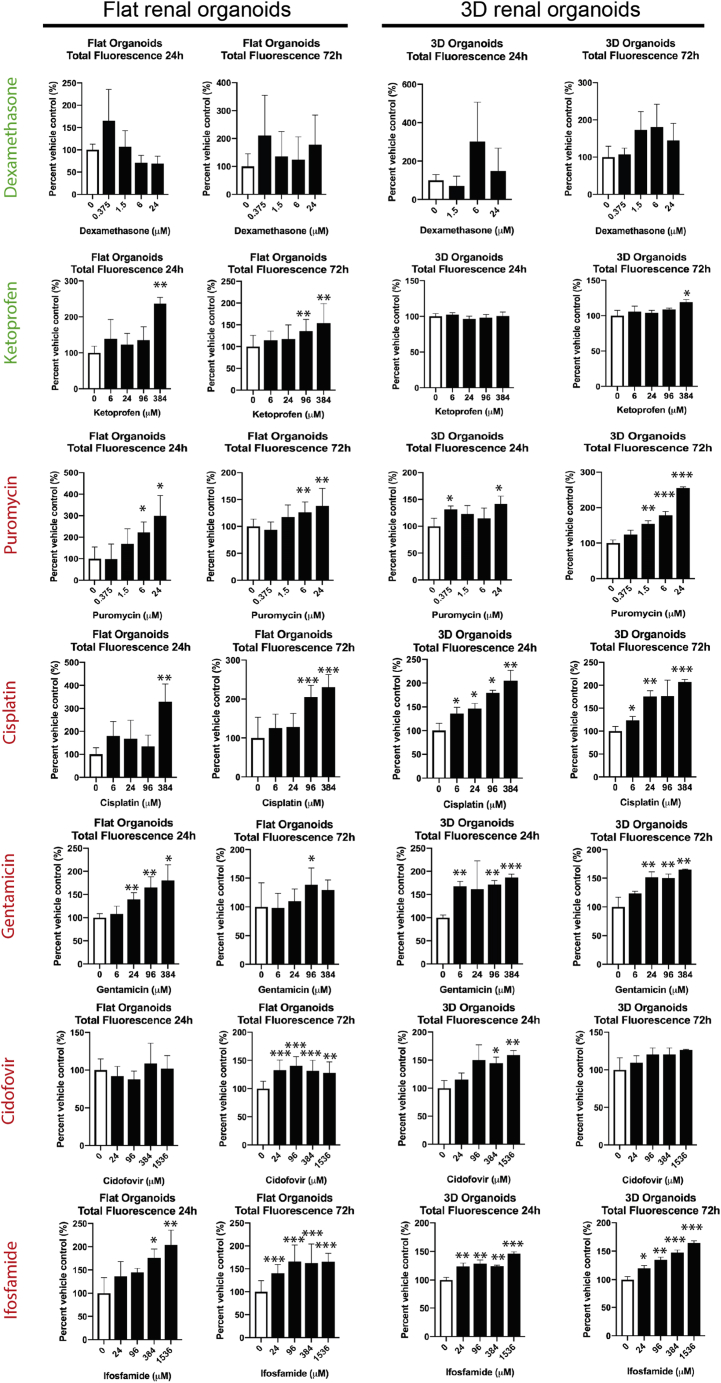
Response of HO1-mCherry-hiPSC-derived flat and 3D organoids to blinded compound screen Blind-coded compounds were used to treat *H O 1*-mCherry-hiPSC-derived renal organoids in either flat (left two panels) or 3D (right two panels) organoid forms, for either 24 h or 72 h, followed by measurements of fluorescence. Results are total fluorescence as a percent of the vehicle control. Significance of fluorescence intensity increase compared to the vehicle control was assessed using a T-test with Welch's correction. Each time point and organoid format (flat or 3D) was carried out as a separate experiment and each included a vehicle-only control. Data are represented as mean ± SEM, p < 0.05 is indicated with (∗), p < 0.01 with (∗∗) and p < 0.001 with (∗∗∗).

*H O 1*-mCherry-hiPSC renal organoids (both flat and 3D) were incubated in a 4-fold dilution series of each compound for either 24 h or 72 h. Data on reporter expression are shown graphically in Figure 5. For dexamethasone, while there was some noise in the data, none of the concentrations produced a signal statistically different (p < 0.05) from other concentrations (including zero), in either the flat or 3D cultures. Thus, the reporter cells correctly identified dexamethasone as non-toxic. The gastrointestinal toxicant ketoprofen induced a significant response in the flat cultures at the two highest concentrations, and in the 3D cultures only at the highest concentration after 72 h.

For puromycin, both flat and 3D organoids showed a significant rise in fluorescence after both 24 and 72 h, with a stronger response after 72 h. Cisplatin induced a significant increase in fluorescence, both in flat and 3D organoids. Again, the 3D organoids responded to lower concentrations than the flat ones, and changes were more evident after 72 h than after 24 h. For gentamicin (which was the toxicant we used to identify *HMOX1* as a potential reporter), the 24 h-treated organoids of both flat and 3D types responded to even low concentrations (6 μM for 3D organoids, 2 4μM for flat organoids). For this compound, the 24 h time point was more informative than 72 h. Ifosfamide induced a significant fluorescence increase in both flat and 3D organoids, at both 24 and 72 h. After 24 h, 3D organoids were more sensitive than flat organoids but, at 72 h, the flat organoids were more sensitive predictors of toxicity. Organoids did not predict cidofovir toxicity as reliably as for the other compounds. Flat cultures treated for 24 h showed no significant response, while treatment for 72 h elicited a response at even the lowest concentration tested. The 3D cultures, however, showed a response after 24 h treatment but not after 72 h.

**Figure 6:**
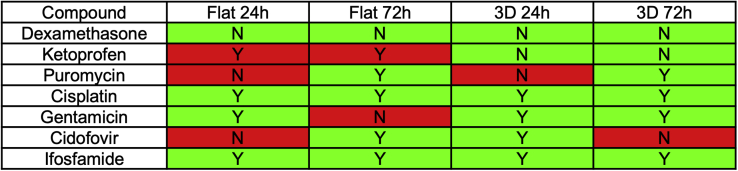
Prediction of toxicity by increase in fluorescence intensity N = Did not predict toxicity, Y = Did predict toxicity. Green indicates results that are consistent with known clinical data. Prediction score is based on data in Figure 4. A p value of 0.01 or more for one or more of the treatment concentrations is designated as having predicted toxicity

Taking successful prediction of a toxin to be response at p < 0.01, and successful prediction of non-toxic nature to be absence of a response even at p < 0.05, the success of the *H O 1*-mCherry-hiPSC-derived organoids at predicting toxicity is summarized in Figure 6. As can be seen from Figure 6, no single combination of timing or organoid shape was completely reliable at prediction. Using a combination of 24 and 72 h and taking a Boolean OR of the results (where any positive data point is considered to mean an overall “positive”), the organoids correctly predicted toxicity for all nephrotoxic compounds. The 3D organoids also did not predict toxicity for either the non-toxic dexamethasone or the GI oxidative stress-induced ketoprofen, unlike the flat organoids that did predict toxicity for ketoprofen at the highest concentrations.

## Discussion

The purpose of this study was primarily to find a reporter of toxicity that could be engineered into human pluripotent stem cells, and then to assess the performance of that reporter using a blind-coded panel of test compounds. In our RNA-seq analyses, classical markers of nephrotoxicity were either not up-regulated in response to injury (*CLU*) or were downregulated (*NGAL*) (data not shown) in response to the known nephrotoxicant gentamicin. Although *KIM1*, a classical *in vivo* renal injury marker, was weakly upregulated in the RNA-seq analysis, reports on its suitability for *in vitro* nephrotoxicity screening in cell culture models are variable (Adler et al., 2016; Li et al., 2013; Huang et al., 2015). Conversely, Alder et al. also found that *HMOX1* was a much more sensitive marker for predicting nephrotoxicity in 2D and 3D microfluidic HPTEC cells. The renal organoids most closely resemble human embryonic kidney tissue as shown by the authors of the protocol (Takasato et al., 2015, 2016), providing an explanation for the lack of induction of classical injury markers such as Kim-1 following injury. An advantage of targeting immediate responses to stress is that even immature tissues will respond. Based on the intersection of *HMOX1* in several different toxicity pathways as well as its greater level of upregulation, we identified *HMOX1* as a potentially promising reporter for one large class of nephrotoxicants, those in which the OS pathway is activated. We engineered the gene for the fluorescent protein mCherry into the locus of *HMOX1* in hiPSCs and showed that organoids made from those hiPSCs can predict toxicity, at least when used with a range of concentrations and measurements done at more than one time point. It is important to note that our assay, using renal organoids alone, does not discriminate between a toxicant dangerous only to kidneys, and one dangerous to other tissues as well.

In our trial of the *H O 1*-mCherry-hiPSC-derived organoids, there was a trend for 3D organoids to be more sensitive than flat ones (that is, to respond to lower concentrations of test compounds). This difference was especially marked for puromycin and cisplatin. *In vivo*, puromycin primarily affects the glomerular/mesangial cells, with significant nephrotoxicity *in vivo* thought to be caused by excessive protein reuptake secondary to serum protein leakage through damaged glomerular filters (Hjalmarsson et al., 2001; Abbate et al., 2006). The organoids, being devoid of flowing blood, cannot flood nephrons with intraluminal protein, and therefore cannot produce this secondary damage. Damage to organoids is mainly expected to be via the primary targets (cells of the renal corpuscle including podocytes and mesangial cells). The extra sensitivity of 3D organoids to puromycin may well reflect the higher proportion of cells associated with the glomeruli such as podocytes in these compared to flat organoids (Takasato et al., 2016; Hale et al., 2018). Cisplatin is taken up mainly through the transporter OCT2 (SLC22A2). As presented in this paper, expression of this transporter was weak in flat organoids and strong in 3D organoids, providing a possible explanation for the increased sensitivity of the latter to cisplatin. Ifosfamide is also taken up by the renal proximal tubule cells through the OCT2 (SLC22A2) transporter (Ciarimboli et al., 2011); however, it is not possible to exclude its uptake by other cation transporters from that study. Ifosfamide may be taken up by other cation transporters such as OCTN1 that are present in the flat organoids, because we see a significant increase in fluorescence with this compound in those organoids. Nevertheless, the more robust increase in fluorescence in the 3D organoids for this compound after 24 h probably reflects the involvement of OCT2 in the uptake of this compound.

Unlike the transporter-driven uptake of cisplatin and ifosfamide, gentamicin is taken up into proximal tubule cells through the combined actions of the endocytic proteins megalin and cubulin. Our RNA-seq analysis showed that these genes are expressed in flat organoids, and thus it is perhaps not surprising that the flat organoids as well as the 3D organoids were able to predict toxicity to this compound. The gentamicin-treated 3D organoids (but not flat ones) maintained their tubular structures in sharp contrast to the cisplatin-treated 3D organoids, in which distinct loss of tubular structure was observed especially at the higher concentrations (data not shown).

When flat and 3D organoids were treated with cidofovir, an increase in fluorescence was observed in flat organoids only after the longer incubation time of 72 h. Cidofovir is taken up in the proximal tubule cells by organic anion transporters OAT1 and, to some extent, OAT3 (Uwai et al., 2007). The low levels of these transporters in the flat organoids might explain the lack of fluorescence at the 24 h time point, with increasing fluorescence only after a longer incubation as is seen with cisplatin. Cidofovir causes damage to tubular cells leading to induction of apoptosis, and *in vitro* studies typically require treatment of several days to induce damage (Ho et al., 2000; Ortiz et al., 2005). Interestingly, the 3D organoids appeared to be relatively insensitive to the compound in terms of induction of oxidative stress and reporter expression, despite the presence of the anion uptake transporter OAT1. Because cidofovir is more selectively toxic to proximal tubules than some of the other compounds tested, it may be that the lack of sensitivity of the 3D renal organoids to cidofovir reflects a more mixed combination of renal cell types, with fewer of them being of proximal tubule identity. The mechanism of cidofovir nephrotoxicity is currently unclear, and it is also important to note that both the flat and 3D renal organoids are closer to human embryonic kidneys than adult kidneys; this immaturity may account for some of the differences between responses. It is also of note that the single endpoint for this study (the fluorescence analysis of oxidative stress reporter) may not identify all toxicants, and that a combination of this assay and another endpoint may be more informative.

We conclude that the reporter-hiPSC-derived renal organoids can predict toxicity to kidneys (Figure 6), but that they are variable in their individual responses to compound treatment and that certain organoid formats (flat or 3D) may be better suited to different classes of drugs. Although the 3D organoids in general could predict toxicity at a lower concentration than the flat organoids, the response for the 3D organoids was generally slower than the flat organoids when assessing the 24 h time point. This may reflect a difference in penetration of compounds in the 3D, dome-shaped organoids with their more densely packed renal structures compared to the flat organoids. In addition, some compounds required longer incubation to elicit a measurable response via the oxidative stress pathway, which may be explained by the different mechanisms of toxicity of these compounds. The inherent variability of the organoids may also play a part in these results and addressing this will be important for the future of organoids for high-throughput drug screening.

The fluorescence increases in response to application of varying concentrations of nephrotoxicants in these organoids require a binary interpretation. These organoids represent a possible broad screening tool for nephrotoxic compounds, and further investigations may help to explain some differences in compound responses and be an additional useful tool to identify mechanisms of toxicity.

Comparison of the *H O 1-*mCherry-hiPSC reporter fluorescence, to qPCR analyses of *HMOX1* transcription in response to the same compounds (Figure S5 and Table S3), shows that there is broad agreement between the data. As mentioned previously, while the increases in fluorescence intensity and the transcript increases of *HMOX1* did not mirror each other exactly, this is likely due to the accumulation of fluorophore with a longer half-life (24 h) (Snapp, 2009; Shaner et al., 2004) than the half-life of the transcripts. The fluorescence assay may provide a more sensitive response than qPCR for predicting toxicity and, additionally provides the option of multiple time points on the same samples as well as using fewer reagents and being less time-consuming.

### Limitations of the study

It is important to note that, while this system could identify toxicities, we do not claim on the strength of the data presented here that it will perform adequately on all possible nephrotoxicants. Rather, we propose it as one useful tool in screening, not as the only system needed. We also stress that a much larger set of compounds (both nephrotoxic and non-nephrotoxic) would have to be tested to validate the system and conclude on its predictive power. Our testing of seven compounds and getting 7/7 correct calls (calls made when considering both flat and 3D organoids together, and regarding a signal in either as an indication of toxicity as explained in the last section of results) is adequate as a proof of concept for engineering reporters into hiPSCs for nephrotoxicity testing, but this modest number of test compounds does not indicate that our test can be relied upon to be correct 100% of the time. The Wilson Score Interval method of calculating CIs indicates that 7/7 correct calls provides 95% confidence of making a wrong call in no more than 44% of tests. The test cannot therefore be relied upon in real screening programs until it has been subjected to a much larger number of tests (by the Wilson Score Interval method, 94/94 correct calls would be needed to give a test a 95% confidence of making a false call in fewer than 5% of tests).

The differentiation of iPSC into organoids always carries the risk of producing off-target cells, neurons being particularly common contaminants in the case of kidney organoids. This raises the possibility of “false-positive” indications of nephrotoxicity if the cells that express HMOX1 are of an off-target type, if cultures are read by simple machine-assessment of fluorescence rather than by microscopic observation. In the context of the intended application of this system—screening candidate compounds for toxicity that would see them fail clinical trials—“false positivity” would not be a major problem because safe compounds should not cause severe stress to any cell type. If neurons, for example, in a renal culture, gave a “false-positive” because in fact the compound was toxic to brain rather than kidney, the overall effect would still be that a potentially dangerous compound was detected before it went forward and caused problems.

The system we describe gives no direct indication of mechanism beyond the presence of oxidative stress somewhere in the organoid. This is sufficient for giving a warning about a test compound, but is of limited help in guiding researchers toward possible strategies for mitigation, either through chemical modification of the compound or co-administration of an uptake-channel inhibitor. These steps would be assisted by establishing which cells of the kidney are (worst) affected by the toxicant. This could be done by sectioning and staining the organoids (which are rather too thick for clear imaging as they are) for HMOX1 and cell- or segment-specific markers. It could also be done by disaggregating the organoid, separating its cells by FACS, and analyzing each population for specific types of damage or even to a complete transcriptomic study if the importance of the test compound warranted that investment. However, given the limitations of organoids in terms of maturity, cultured primary cells or tissues (for example, from potential transplant kidney that could not be used) would probably be a much more informative system in which to follow up mechanisms of toxicity of a compound worth the effort, once toxicity had been indicated by the simple screen described in this report. We are not in any way claiming that the system described here can yield high-resolution mechanistic data; rather, that it can provide a simple indication of potential danger (to offer an analogy: it is a smoke alarm, not a mass spectrometer).

### Conclusions

Subject to the limitations discussed in the forgoing section, we have demonstrated an effective organoid-based test for nephrotoxicity by oxidative stress that features an easy to read, built-in fluorescent reporter system.

Although we differentiated our cells toward the renal lineage, as a global marker of oxidative stress, a reporter for *HMOX1* expression in human pluripotent cells will be an invaluable tool for a broad range of applications in other organoids and engineered tissues.

## STAR★Methods

### Key resources table

**Table:** 

REAGENT or RESOURCE	SOURCE	IDENTIFIER
**Antibodies and lectins**		

Mouse anti-OCT3/4	BD Biosciences	611202; RRID: AB_398736
Nanog	Abcam	Ab62734; RRID: AB_956161
Rabbit anti-Brachyury	Santa Cruz Biotechnology	sc-20109; RRID: AB_2255702
Goat anti-LHX1	Santa Cruz Biotechnology	sc-19341; RRID: AB_2135492
Rabbit anti-PAX2	Biolegend	PRB-276-200
Rabbit anti-WT1	Santa Cruz Biotechnology	sc-192; RRID: AB_632611
Mouse anti-CDH1	BD Bioscience	610181; RRID: AB_397580
Goat anti-JAG1	R&D Systems	AF599; RRID: AB_2128257
Sheep anti-NPHS1	R&D Systems	AF4269; RRID: AB_2154851
Mouse anti-HMOX1	Invitrogen	MA1-112; RRID: AB_2536823
Rabbit anti-mCherry	Novus	NBP2-25157SS
Fluorescein-conjugated LTL	Vector Laboratories	FL-1321-2
Biotin-conjugated LTL	Vector Laboratories	B-1325
Mouse anti-CALB	Abcam	Ab75524; RRID: AB_1310017
Goat anti-GATA3	R&D Systems	AF2605; RRID: AB_2108571
Donkey anti-mouse Alexa Fluor 488	Thermo-Fisher	A21202; RRID: AB_141607
Donkey anti-rabbit Alexa- 488	Thermo-Fisher	A21206; RRID: AB_2535792
Donkey anti-goat Alexa- 488	Thermo-Fisher	A11055; RRID: AB_2534102
Donkey anti-mouse Alexa Fluor 594	Thermo-Fisher	A21203; RRID: AB_141633
Donkey anti-rabbit Alexa Fluor 594	Thermo-Fisher	A21207; RRID: AB_141637
Donkey anti-mouse Alexa Fluor 350	Thermo-Fisher	A10035; RRID: AB_2757556
Donkey anti-rabbit Alexa Fluor 647	Thermo-Fisher	A31573; RRID: AB_2536183
Donkey anti-sheep Alexa Fluor 488	Thermo-Fisher	A11015; RRID: AB_141362
Streptavidin-conjugated Alexa Fluor 350	Thermo-Fisher	S11249

**Chemicals, peptides, and recombinant proteins**		

Matrigel	Corning	354277
Essential 8 (E8) medium	Gibco	A1517001
ROCK inhibitor Y-27632	Tocris	1254
EDTA	Fluka	03690
APEL2 culture medium	Stemcell Technologies	05270
CHIR99201	Tocris	4423-10mg
Recombinant human FGF9	R&D Systems	273-F9-025
Heparin	Stemcell Technologies	07980
Trypsin/ EDTA	Sigma-Aldrich	SLCB0546
Hydrogen peroxide to induce oxidative stress	Fisher Scientific	BP2633-500
Probenecid	Sigma-Aldrich	P8761
6-Carboxyfluorescein	Invitrogen	C1360
Rhodamine-conjugated peanut agglutinin	Vector Laboratories	RL-1072
4-(4-(dimehylaminostyryl))-N-methylpyridinium iodide (ASP)	Invitrogen	D288
Tetrapentyl ammonium chloride (TPA)	Sigma-Aldrich	258962
Gentamicin	Sigma-Aldrich	G1397
Gentamicin	Sigma-Aldrich	G1264
Cisplatin	Tocris	2251
Cisplatin	Sigma-Aldrich	C2210000
Cidofovir	Sigma-Aldrich	C5874
Dexamethasone	Sigma-Aldrich	D1756
Ifosfamide	Sigma-Aldrich	I4909
Ketoprofen	Sigma-Aldrich	K1751
Puromycin	Sigma-Aldrich	P7255

**Critical commercial assays**		

Alkaline phosphatase detection kit	Merk Millipore	SCR004
RNeasy mini kit	Qiagen	74014
TaqMan Fast Universal PCR Master Mix (2X)	Applied Biosystems, Thermo Fisher	4352042
Illumina TruSeq stranded mRNA kit	Illumina	20020594

**Deposited data**		

RNA-seq data	This paper	GEO accession number GSE195642
Image sets from the different experiments	This paper	DataShare@ed.ac.uk

**Experimental models: Cell lines**		

hiPSCs	Lonza	clone AD3-01
*HO1*-mCherry-hiPSCs	This paper	N/A

**Oligonucleotides**		

See Table S5 for primer sequences	N/A	N/A
See Table S5 for gRNA sequences	N/A	N/A
HMOX1 probe	Applied Biosystems	Hs01110250_m1
HPRT Probe	Applied Biosystems	Hs99999909_m1
RPLP0 probe	Applied Biosystems	Hs99999902_m1

**Software and algorithms**		

fiji-image J		https://fiji.sc/
GraphPad Prism version 8.0.0	GraphPad Software, San Diego, California USA	www.graphpad.com
gRNA design tool	Zhang lab resource	crispr.mit.edu
Cutadapt	National Bioinformatics Infrastructure Sweden	version cutadapt-1.9.dev2
STAR		version 2.5.2b
Feature Counts		version 1.5.1
PANTHER	Panther classification system	http://pantherdb.org/ release date 2018-10-08
edgeR4	Bioconductor	version 3.20.1

**Other**		

Transwell inserts	Scientific Laboratory Supplies	3450
pUC18 (vector plasmid)	Addgene	50004
pCas9_GFP	Addgene	44719

### Resource availability

#### Lead contact

Further information and requests for resources and reagents should be directed to and will be fulfilled by the lead contact, Jamie Davies (jamie.davies@ed.ac.uk).

#### Materials availability

The HO1-mCherry-hiPSC oxidative stress reporter cell line generated in this study is available in Jamie Davies' lab, Edinburgh University, and can be obtained upon request.

### Experimental model and subject details

#### hiPSC line and maintenance

hiPS cells (clone AD3-01, derived from adult human dermal fibroblasts) were plated on Matrigel-coated plates and cultured in Essential 8 (E8) medium at 37°C in 5% CO_2_. When they had reached 80% confluence, cells were treated for 1 hour with the ROCK inhibitor Y-27632 (10μM) then were dissociated in 0.5 mM EDTA in PBS and plated on Matrigel-coated plates. The culture medium was supplemented with ROCK inhibitor (10μM) for the first 24 hours. Media were changed daily, with changes having no ROCK inhibitor.

### Method details

#### Production of hiPSC-derived renal organoids

Pluripotency was affirmed in our starting population of hiPSCs by immunostaining for the pluripotency markers Oct3/4 and Nanog, and positive staining for Alkaline Phosphatase (Figure S1A). An established protocol (Takasato et al., 2015), with minor modifications (Figure S1B), was applied to the hiPSCs to induce renal differentiation via a path that differentiates hiPSC first to a primitive streak-like identity, then to an intermediate mesoderm-like identity and finally to the precursors of renal development: ureteric bud and metanephric mesenchyme. After 2 days of CHIR99201 treatment, Oct3/4 expression was replaced by the expression of primitive streak marker, Brachyury (Figure S1C left panel). After a further 2 days of CHIR99201, cells were treated with FGF9 and heparin to induce differentiation towards the intermediate mesoderm (IM) fate. By day 5 of the treatment with FGF9 and heparin the cells expressed LHX1 and PAX2, markers of IM (Figure S1C right panel). After 12 days in total, exogenous FGF9 and heparin were withdrawn and the cells continued to differentiate towards renal fate. By day 18 of differentiation bundles of tubules could be seen in flat organoids, which spread across the bottom of a well (Figure S1D). To make 3D organoids, undifferentiated cells were cultured as above in six-well plates (144,000 cells/well) until 7 days after starting differentiation, when each well was dissociated using Trypsin/EDTA, and pelleted at 300,000 cells/pellet (Figure S1E). Pellets were grown on the surface of transwell inserts with medium added to the lower compartment only. 10μM Y-27632 was added to the culture media for overnight after dissociation and was removed afterwards. The re-aggregated pellets were treated with Heparin (1 μg/mL) and FGF9 (200 ng/mL) for a further 5 days, then were withdrawn and no further growth factors were given. Differentiation was complete by 18 days, and resulted in dome-shaped organoids with robust and dense tubule development (Figure S1F).

#### CRISPR-Cas9 editing of HMOX1 gene in hiPSCs

We designed a targeting vector containing a 2A-peptide-mCherry-STOP cassette flanked by homologous 5′ and -3′ arms upstream (500bp) and downstream (1049bp) of the endogenous stop codon of the endogenous *HMOX1* gene in the hiPSCs (Figure 3A). The targeting sequence was synthesized by Integrated DNA Technologies and then subcloned into pUC18 for transfection. Guide RNAs (gRNAs) were designed using the Zhang lab resource) (Table S4). Undifferentiated hiPSCs were co-transfected in equimolar amounts with the Cas9-GFP (pCas9_GFP; Addgene) and gRNA plasmids together with the targeting vector containing the repair template. After letting the cells recover and proliferate for 72h, cells were treated with hydrogen peroxide to induce oxidative stress (0, 25 or 150μM) and then sorted using FACS to isolate the mCherry-targeted population (Figure S4A). Wild type hiPSCs were used as a control for gating. The enriched populations were recovered and expanded for a further 7 days. To isolate clonal populations of targeted hiPSCs, we again used FACS. The low but detectable basal level of HMOX1 expression in undifferentiated hiPSCs allowed us to sort single, weakly mCherry-positive hiPSCs into wells of a 96-well plate without needing to first induce oxidative stress (Figure S4B). Wild type hiPSCs (AD3-01) were again used for gating. After a further 7 days, 24 clonal populations from the single-cells sorted by FACS had survived and expanded. These were further expanded into individual wells of a 6-well plate. PCR, using primers designed to span the targeted region (Table S4), was used to confirm that the reporter cassette had been integrated (Figure S4C primers 1 and 4). The product size without cassette insertion is 1654bp, whereas product size with insertion is 2428bp (Figure S4D), as expected. Clones were sequenced using primers spanning the homologous arms and inserted cassette (primers 1 and 2 at 5′ end and primers 3 and 4 at 3′ end) (Table S4).

#### RT-PCR and qPCR

Total RNA was isolated from organoids using the Qiagen RNeasy mini kit. Flat organoids were dissociated and processed as per the kit protocol. 3D organoids were lysed using the lysis buffer provided in the kit and then passed through a needle to fully dissociate before continuing with the kit protocol. RNA was stored at −80 °C until use. cDNA for qPCR was prepared using 2μg RNA and cDNA for RT-PCR was prepared using 1μg of RNA. All cDNA was quality-controlled by PCR using intron-spanning primers for β-actin on cDNA, prepared with or without reverse transcriptase (primers in Table S4). RT-PCRs for *OAT1 (SLC22A6)* and *OCT2 (SLC22A2)* were carried out in multiplex reactions with primers for β-actin as loading controls (primers in Table S4). qPCRs on undifferentiated and flat organoids for *HMOX1* with *HPRT1* as internal control were performed using TaqMan reagents as per published protocols (Applied Biosystems, assay nos. in Table S4). For qPCRs in the blind-coded screen, quality-controlled cDNA was provided by the Edinburgh authors to the Gothenburg authors. 12 ng of cDNA was utilized for each qPCR reaction. TaqMan gene expression assays (Applied Biosystems, TaqMan Gene Expression FAST Master Mix) were performed on HMOX1 and house-keeping genes HPRT and RPLP0. The reaction was prepared via Beckman NX and measured on Applied Biosystems QuantStudio 7 Real-Time PCR System.Technical triplicates served to calculate CT mean for each gene. The ΔCT between the target gene and the mean CT of the two reference genes was calculated for each biological replicate. The mean ΔCT of biological triplicates was normalized to untreated sample resulting in 2^ΔΔCT^. The square root of vehicle and treated sample variance resulted in the standard deviation of ΔCT treated sample. A one-way ANOVA on repeated measures was performed. Significant fold-change was established by Dunnett's multiple comparisons test.

#### RNA-seq analysis

RNA-seq, and trimming, filtering and normalization of raw data, was performed by Edinburgh Genomics. 1 μg of total RNA/sample was used for sequencing library preparation using an Illumina TruSeq stranded mRNA kit. Sequencing was performed using a Novaseq S1 50 Paired-end run to produce 50–64 million read-pairs per sample. Reads were trimmed using Cutadapt and were aligned to the *Homo sapiens* reference genome (GRCh38; from Ensembl) using STAR. Standard GTF-format annotation for the reference genome (annotation version 84) was used. Reads were assigned to features of type ‘exon’ in the input annotation grouped by gene_id in the reference genome using Feature Counts. The raw count data were filtered to remove very low-expressed genes (genes consisting predominantly of near-zero counts), filtering was performed on counts per million to avoid artefacts due to library depth. Reads were normalised using the weighted trimmed mean of M-values method. Differential analysis (comparing the low-treated group to the control group, or the high-treated group to the control group) was carried out using edgeR4. Fold-changes were estimated as per the default behavior of edgeR, to avoid artefacts which occur with empirical calculation. Statistical assessment of differential expression was carried out with the quasi-likelihood (QL) F-test.

To analyse the up-regulated genes in the high-treated versus the control contrast, we first selected the 400 genes with the lowest p value corrected for FDR, then from these picked the 100 most strongly up-regulated and used them for downstream analysis. We performed a PANTHER Over-representation Test using the annotation version GO Ontology database. For the functional classification, the same list of genes was used in a Panther functional classification analysis selecting the ontology Biological Process and the ontology Molecular Function.

#### Immunohistochemistry

The culture medium was aspirated, cells washed once in PBS, then fixed in 4% PFA in PBS for 15 minutes at room temperature, followed by three PBS washes of 3 min each. Cells were permeabilised and blocked for 1 hour (2% BSA, 0.3% Triton x100 in PBS). Cells were washed in PBS and incubated with primary antibodies overnight at 4 °C. Cells were washed in PBS (3x, 15 min) and incubated with secondary antibodies for 2 hrs at room temperature. Cells were washed in PBS (3x, 15 minutes) before imaging in PBS in the wells. Images were taken using a Zeiss Axiovert fluorescence microscope. Working dilutions of the antibodies were as follows: anti-HMOX1, 1:100; anti-mCherry, 1:100; anti-Oct3/4, 1:100; anti-Pax2, 1:200; anti-LHX1, 1:100; anti-CALB, 1:200; anti-WT1, 1:200; anti-CDH1, 1:300; anti-NPHS1, 1:200; anti-BRACHYURY, 1:100. Details of the used antibodies are listed in the key resources table. Antibody solutions were prepared using PBS containing 1% BSA.

#### Transporter assays

The transporter assay for organic anion uptake on 20-day 3D renal organoids were performed using the method of (Lawrence et al., 2015). Probenecid was purchased from Sigma-Aldrich, 6-carboxyfluorescein was purchased from Invitrogen (6-CF) and Rhodamine-conjugated peanut agglutinin was purchased from Vector Laboratories. 6-carboxyfluorescein was solubilised in water to make a stock concentration of 1 mM. Probenecid was solubilised in 500 mM NaOH, to make a stock concentration of 250 mM. Reagents for live assays were diluted to their final concentrations in APEL culture medium. The transporter assay for organic cation uptake was performed as follows: 20-day differentiated organoids were incubated overnight with biotin-conjugated LTL (final concentration 20 μg/mL in APEL medium) to label the proximal tubules. After washing, the organoids were incubated for 1 h at 37 °C with streptavidin-conjugated Alexa Fluor 350 (final concentration 10 μg/mL in culture medium). After a further wash, organoids were incubated with 4-(4-(dimehylaminostyryl))-N-methylpyridinium iodide (Asp+) for 30 minutes. For the inhibitor-treated organoids, the OCT2 inhibitor tetrapentyl ammonium chloride (TPA) (final concentration 1 mM) was incubated with the organoids for 10 minutes before addition of the Asp+ (still in the presence of TPA), and incubating for a further 30 minutes. After incubation organoids in both assays were washed in ice-cold PBS and transferred to a microscope slide, covered with one drop of ice-cold PBS and imaged immediately.

#### Verifying HO1-mCherry-hiPSC stress response

Gentamicin was purchased from Sigma-Aldrich (G1397) at a stock concentration of 50 mg/mL. Cisplatin was purchased from Tocris (2,251) and was diluted in phosphate-buffered saline (PBS) to make a stock concentration of 100μM. Compounds were diluted in culture medium (E8) at the appropriate concentrations to treat the flat renal organoids. Flat renal organoids were cultured in 24-well plates.

#### Blind-coded screen

A panel of 7 compounds for the blind-coded validation, selected and supplied by the Gothenburg authors without revealing their identities to the Edinburgh authors, was as follows: Cidofovir, Cisplatin, Dexamethasone, Gentamicin, Ifosfamide, Ketoprofen and Puromycin; all from Sigma-Aldrich. The compounds were divided into 3 groups based on their therapeutic Cmax values (Sjögren et al., 2018) and concentration ranges were designed accordingly: 0.375–24 μM for Dexamethasone and Puromycin, 6–384 μM for Cisplatin, Gentamicin and Ketoprofen, 24–1536 μM for Cidofovir and Ifosfamide. A four-fold dilution series of each blinded compound (lyophilized, obtained from the Gothenburg authors) was prepared and HO1*-*mCherry-hiPSC-derived renal organoids (flat and 3D) were incubated in these dilutions for either 24h or 72h (Figure 5). A no-treatment control was included for each compound, time-point and organoid type (flat or 3D). 24h flat organoids were cultured in 4 x wells of a 24-well plate; 72h flat organoids in wells of a 96-well plate and 3D organoids on transwell inserts.

### Quantification and statistical analysis

#### Fluorescence measurement

Fluorescence measurements were taken after 24h or 72h. Each live organoid was imaged either 5 times with 4 replicates (flat organoids, 24h), once with 10 replicates (flat organoids, 72h), once with 3 replicates (3D organoids, 24h) or 3 times with 3 replicates (3D organoids, 72h). Organoids were imaged using a Zeiss Axiovert microscope with Axiovision software. All images used for fluorescence intensity analysis were taken with identical exposures and settings. All images were analysed using ImageJ. The total fluorescence intensity of the image was multiplied by the total area with a signal between 600 (lower threshold) and 4096 (higher threshold), where 0 is completely black and 4096 is completely white. Thus, only signal above a certain threshold is measured in order to discount very dark areas with no cells.

#### Statistical analysis

Fluorescence intensity measurements for each concentration of each compound were compared to their respective untreated measurements using Welch's unequal variances t-test (unpaired) with a null hypothesis that there is no significant difference between the experiment and the control. In graphs, a single star indicates a p value of the null hypothesis being true of less than 0.05, two stars a p value of less than 0.01 and 3 stars a p value of less than 0.001. Calculation of the overall performance of the assay (in the Discussion section) was performed by calculating the Wilson Score Interval method (Newcombe, 1998).

## Data Availability

• RNA-seq data are publicly available in GEO (GSE195642) • This study did not generate novel computer code/software • Image sets will appear on acceptance at DataShare@ed.ac.uk and will be publicly available and can be located by searching on the title of this paper. • Any additional information required to reanalyze the data reported in this paper is available from the lead contact upon request.
