# Supplementary material for: Human iPSC-derived renal organoids engineered to report oxidative stress can predict drug-induced toxicity
Source: iScience. 2022 Feb 7;25(3):103884. doi: 10.1016/j.isci.2022.103884 (PMC8861638; doi:10.1016/j.isci.2022.103884)
Supplement: Document S1. Figures S1–S5 and Tables S1–S5 [file mmc1.pdf]

iScience, Volume *25*

Supplemental information

Human iPSC-derived renal organoids

engineered to report oxidative stress

can predict drug-induced toxicity

M.L. Lawrence, M. Elhendawi, M. Morlock, W. Liu, S. Liu, A. Palakkan, L.F. Seidl, P. Hohenstein, A.K. Sjögren, and J.A. Davies

Figure S1. Validation of differentiation of wild-type hiPSC-derived renal organoids, Related to Figure 1. (Legend appears on the next page).

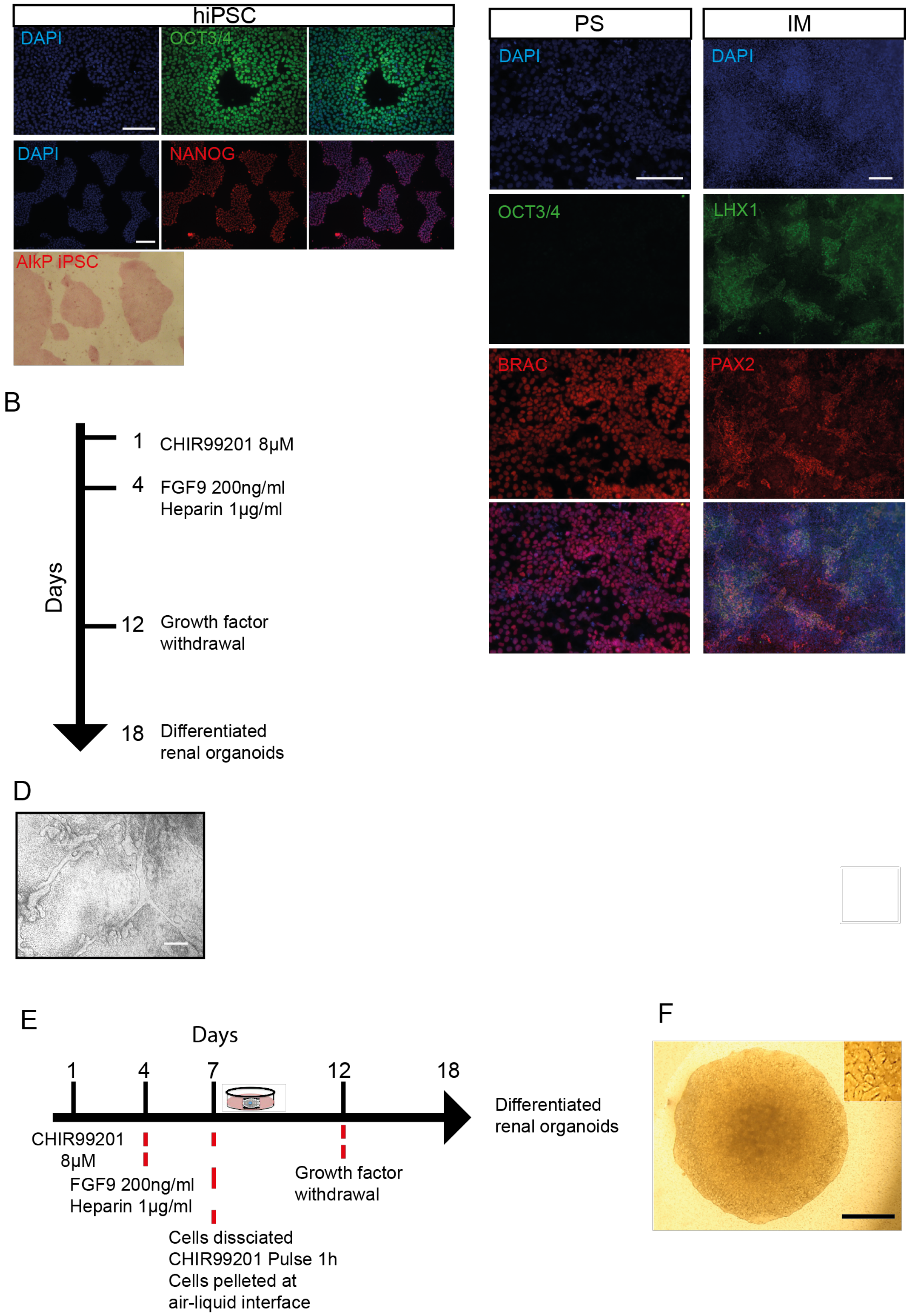

A) Undifferentiated hiPSCs express the markers of pluripotency hOCT3/4 (green, top panels) and hNANOG (red, middle panels) and are positive for the alkaline phosphatase assay (lower panel). B) Differentiation protocol used for induction of flat renal organoids (Takasato et al., 2015). C) hiPSCs lose OCT3/4 expression and express the primitive streak (PS) marker Brachyury (red) after 2 days in culture (left panel PS), and express the markers of intermediate mesoderm (IM) PAX2 (red) and LHX1 (green) (right panel IM) after 9 days. D) Brightfield image of a flat renal organoid. E) Differentiation protocol for induction of 3D renal organoids. F) Brightfield image of renal organoid in a 3D (dome-shaped) format. Scale bars are 100μm (1A-E) or 1mm (1F).

Figure S2. RNA-seq expression analyses for various transporters in wild type, untreated hiPSC-derived flat renal organoids, Related to Figure 1.

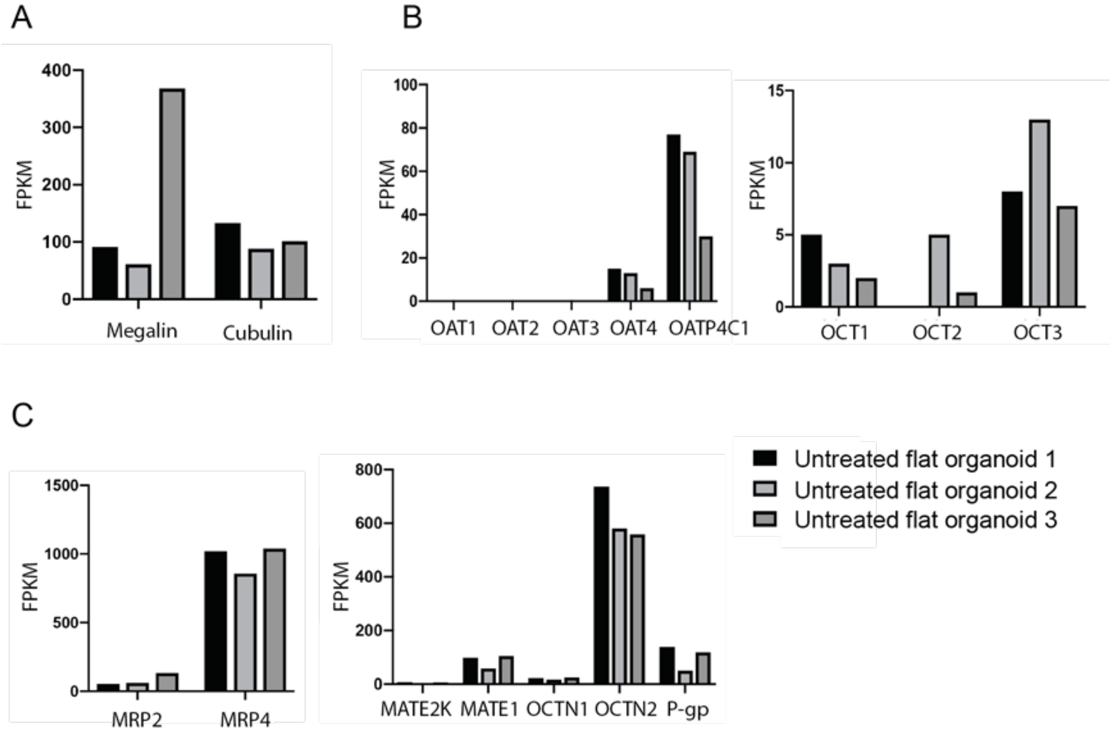

Expression of *Megalin* and *Cubulin* (A), organic anion and cation uptake transporters (B) and efflux transporters (C) in untreated hiPSC-derived flat renal organoids by RNA-seq transcript analyses. FPKM: fragments per kilobase of transcript per Million mapped reads. Transcript analyses for each of the three untreated organoids are shown (Organoids 1-3 in legend).

Figure S3. HMOX1 expression in 3D organoids in response to nephrotoxicants, Related to Figure 2.

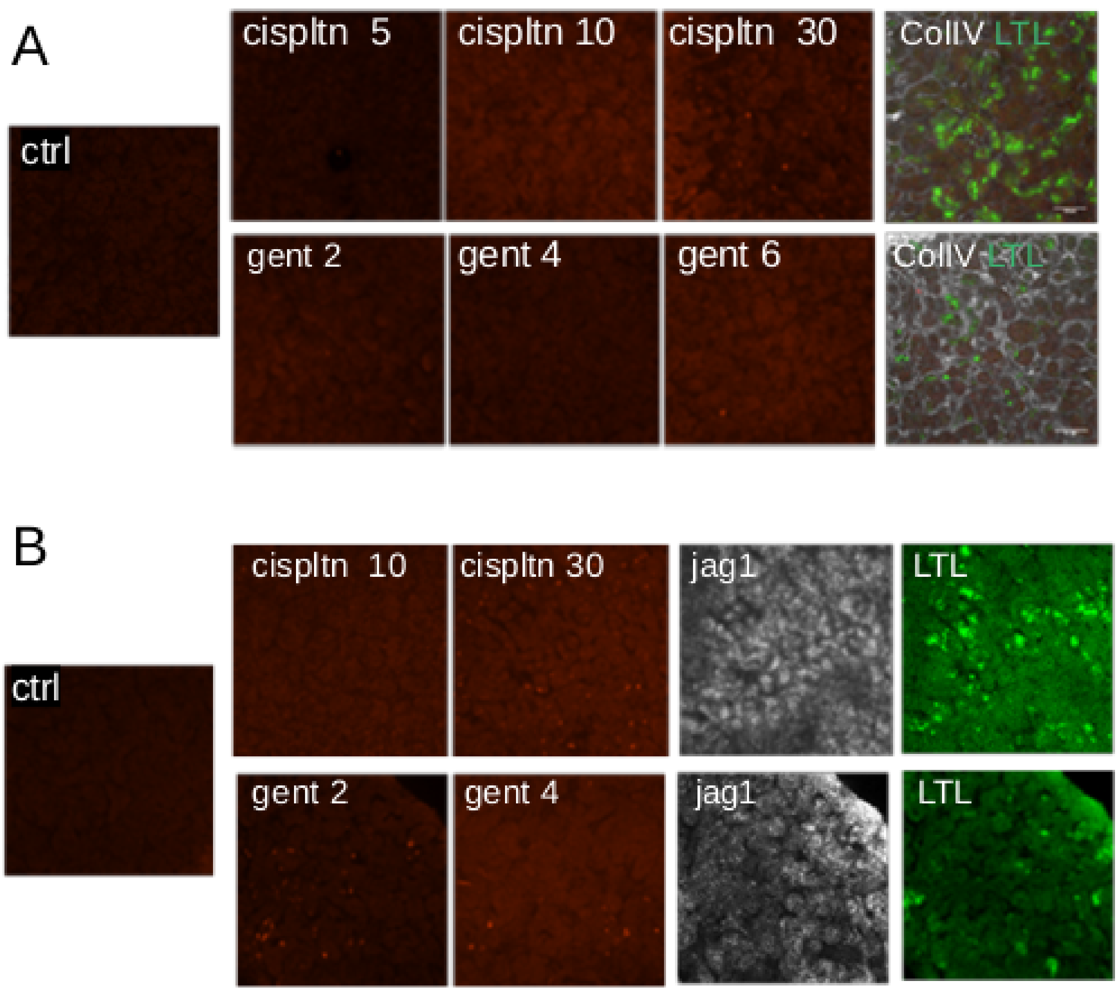

3D organoids are treated with either cisplatin or gentamicin at the concentrations stated in each micrograph (µM for cisplatin, mg/ml for gentamicin): each HMOX expression micrograph was taken at a constant exposure within Fig S3A, and a constant exposure within Fig S3B. Treated samples were also stained for LTL and COL IV (A), or LTL and JAG1 (B). The organoids show increase in HMOX1 expression in response to the toxicant treatment, but ubiquitously, with no apparent concentration of response in proximal tubules or any other component. Scale bar (on top right image) 50µm.

Figure S4. Strategy and validation of reporter cassette insertion into hiPSCs, Related to Figure 3.

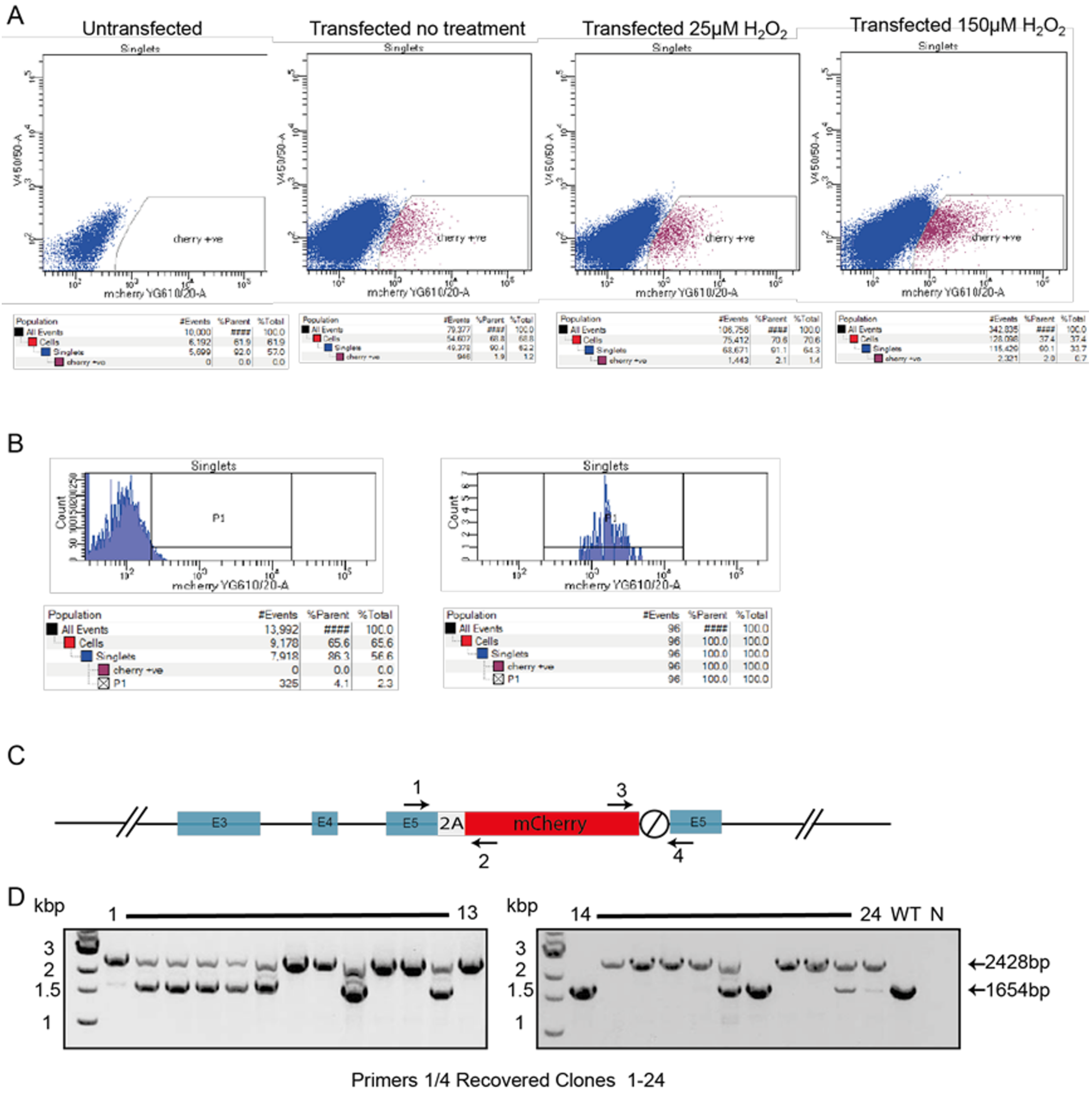

Targeting strategy and clone isolation for *HO1*-mCherry-hiPSC reporter lines. A) Enrichment of targeted cells by fluorescence activated cell sorting (FACS) of either un-transfected (wt) hiPSCs, or CRISPR targeted hiPSCs with 0, 25 or 150µM hydrogen peroxide to induce expression of *HMOX1* through induction of oxidative stress. Basal *HMOX1* expression allowed recovery of cells containing the 2A-

mCherry insertion without hydrogen peroxide treatment. B) Single mCherry-positive cells were sorted into each well of 96-well plates as shown in the right panel to isolate clonal populations. Negative (wild type) cells are shown in the left panel. C) Diagram of the primers designed for verifying cassette insertion into the hiPSC genome. D) PCR products using primer pair 1 and 4 shown in C) - endogenous allele 1654bp, with insertion 2428bp.

Figure S5. *HMOX1* expression in flat and 3D *HO1*-mCherry-hiPSC-derived flat and 3D organoids by qPCR fold-change analysis, Related to Figure 6.

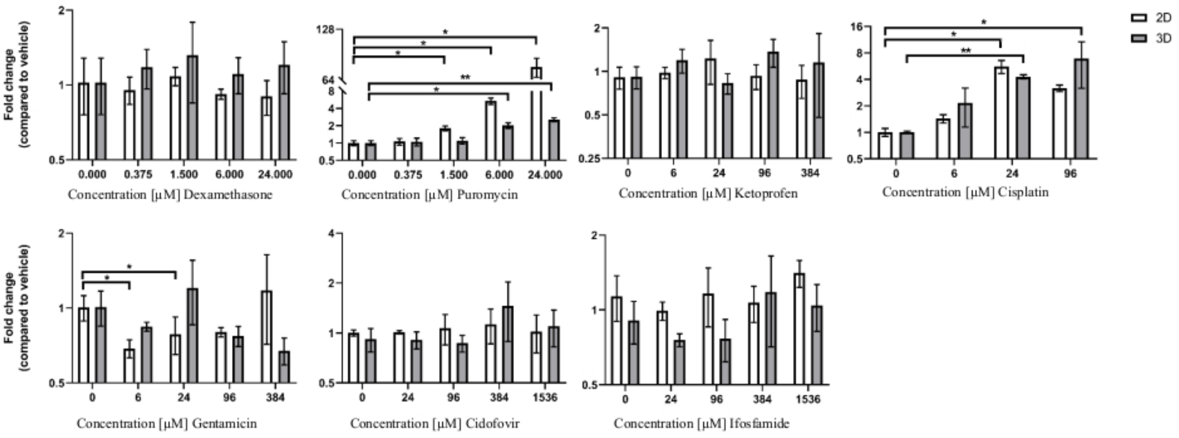

HMOX1 expression fold-change compared to vehicle control in flat (white) and 3D (grey) organoids after 24h treatment with Dexamethasone, Puromycin, Ketoprofen, Cisplatin, Gentamicin, Cidofovir, Ifosfamide, n = 3, ±SD, * < 0.05, ** < 0.01. Cisplatin: no cDNA obtained for highest concentration (384µM), due to massive cell death.

Table S1. Expression of stress-related genes in gentamicin-treated wild type hiPSC-derived flat renal organoids, Related to Figure 2.

Gene Name	Log_2FC	FDR (False Discovery Rate)
LTA	3.873178046	0.000126499
HSPA1B	2.886613617	3.26E-05
JDP2	1.881787141	7.10E-05
HSPA1A	2.663259915	1.75E-05
HSPA6	5.205504565	3.52E-05
ATF3	3.080254651	0.000376849
FOS	3.870520570	0.000598260
JUN	1.372582654	0.022640799
HRK	3.327710653	0.001257237
KIM1	1.669097501	0.031340852
HMOX1	6.552266071	0.000147085

False discovery rate (FDR) 0.05; Log fold-change cut-off 2. The list is not exhaustive and all details can be found in the archived experimental data in the University of Edinburgh database (https://datashare.is.ed.ac.uk/).

Table S2. Analysis of RNA-seq data by GO-term, Related to Figure 2.

GO biological process term	# ref genes in category	GO-term dist 100 top genes	Expected based on chance	Fold enrichment	Significance (p value)	Significance with FDR adjustment
detoxification of inorganic compound (GO:0061687)	15	9	0.08	> 100	2.08E-15	3.27E-11
detoxification of copper ion (GO:0010273)	14	8	0.07	> 100	1.08E-13	4.25E-10
stress response to copper ion (GO:1990169)	14	8	0.07	> 100	1.08E-13	3.40E-10
stress response to metal ion (GO:0097501)	16	9	0.08	> 100	3.23E-15	2.54E-11
cellular response to zinc ion (GO:0071294)	22	8	0.11	70.57	1.91E-12	3.01E-09
negative regulation of inclusion body assembly (GO:0090084)	11	4	0.06	70.57	8.50E-07	3.43E-04
cellular response to copper ion (GO:0071280)	26	8	0.13	59.71	5.83E-12	7.65E-09
cellular zinc ion homeostasis (GO:0006882)	31	9	0.16	56.34	4.06E-13	1.06E-09
zinc ion homeostasis (GO:0055069)	34	9	0.18	51.37	8.26E-13	1.63E-09
cellular response to cadmium ion (GO:0071276)	36	9	0.19	48.52	1.29E-12	2.25E-09
PERK-mediated unfolded protein response (GO:0036499)	12	3	0.06	48.52	5.69E-05	1.38E-02
regulation of inclusion body assembly (GO:0090083)	17	4	0.09	45.66	3.64E-06	1.22E-03
response to copper ion (GO:0046688)	41	8	0.21	37.87	1.36E-10	1.43E-07
protein refolding (GO:0042026)	21	4	0.11	36.97	7.57E-06	2.34E-03
chaperone cofactor-dependent protein refolding (GO:0051085)	28	5	0.14	34.66	6.78E-07	2.81E-04
response to zinc ion (GO:0010043)	54	9	0.28	32.35	3.19E-11	3.86E-08
response to cadmium ion (GO:0046686)	62	10	0.32	31.3	3.26E-12	4.66E-09

'de novo' posttranslational protein folding (GO:0051084)	33	5	0.17	29.4	1.40E-06	5.26E-04
positive regulation of erythrocyte differentiation (GO:0045648)	28	4	0.14	27.72	2.09E-05	6.10E-03
chaperone-mediated protein folding (GO:0061077)	56	8	0.29	27.72	1.25E-09	1.10E-06
'de novo' protein folding (GO:0006458)	37	5	0.19	26.23	2.34E-06	8.01E-04
regulation of cellular response to heat (GO:1900034)	45	6	0.23	25.88	2.33E-07	1.11E-04
cellular transition metal ion homeostasis (GO:0046916)	105	13	0.54	24.03	3.37E-14	1.77E-10
transition metal ion homeostasis (GO:0055076)	132	13	0.68	19.11	5.01E-13	1.13E-09
detoxification (GO:0098754)	110	10	0.57	17.64	5.77E-10	5.67E-07
regulation of erythrocyte differentiation (GO:0045646)	44	4	0.23	17.64	1.06E-04	2.43E-02
negative regulation of fat cell differentiation (GO:0045599)	45	4	0.23	17.25	1.15E-04	2.59E-02
positive regulation of blood vessel endothelial cell migration (GO:0043536)	54	4	0.28	14.38	2.23E-04	4.74E-02
response to unfolded protein (GO:0006986)	154	10	0.79	12.6	1.21E-08	9.07E-06
positive regulation of ubiquitin-dependent protein catabolic process (GO:2000060)	94	6	0.48	12.39	1.26E-05	3.74E-03
positive regulation of proteasomal ubiquitin-dependent protein catabolic process (GO:0032436)	81	5	0.42	11.98	8.02E-05	1.91E-02
cellular response to metal ion (GO:0071248)	185	11	0.95	11.54	5.17E-09	4.28E-06
cellular response to unfolded protein (GO:0034620)	118	7	0.61	11.51	3.66E-06	1.20E-03
response to heat (GO:0009408)	104	6	0.54	11.2	2.17E-05	6.11E-03
response to topologically incorrect protein (GO:0035966)	174	10	0.9	11.15	3.63E-08	2.28E-05
negative regulation of growth (GO:0045926)	256	14	1.32	10.61	1.03E-10	1.16E-07
transition metal ion transport (GO:0000041)	110	6	0.57	10.59	2.94E-05	7.83E-03
cellular response to inorganic substance (GO:0071241)	208	11	1.07	10.26	1.64E-08	1.12E-05
cellular response to topologically incorrect protein (GO:0035967)	136	7	0.7	9.99	8.90E-06	2.69E-03
positive regulation of proteolysis involved in cellular protein catabolic process (GO:1903052)	117	6	0.6	9.95	4.09E-05	1.04E-02
positive regulation of proteasomal protein catabolic process (GO:1901800)	102	5	0.53	9.51	2.25E-04	4.72E-02
positive regulation of cellular protein catabolic process (GO:1903364)	137	6	0.71	8.5	9.46E-05	2.19E-02
protein folding (GO:0006457)	208	9	1.07	8.4	1.75E-06	6.41E-04
regulation of ubiquitin-dependent protein catabolic process (GO:2000058)	147	6	0.76	7.92	1.37E-04	3.00E-02
response to metal ion (GO:0010038)	358	13	1.84	7.05	5.52E-08	3.22E-05
response to toxic substance (GO:0009636)	482	17	2.48	6.84	6.16E-10	5.71E-07
cellular metal ion homeostasis (GO:0006875)	532	16	2.74	5.84	1.84E-08	1.21E-05
response to inorganic substance (GO:0010035)	515	14	2.65	5.28	5.11E-07	2.17E-04
cellular cation homeostasis (GO:0030003)	591	16	3.05	5.25	7.58E-08	4.26E-05
cellular divalent inorganic cation homeostasis (GO:0072503)	444	12	2.29	5.25	3.77E-06	1.21E-03
metal ion homeostasis (GO:0055065)	601	16	3.1	5.17	9.48E-08	4.97E-05
cellular ion homeostasis (GO:0006873)	604	16	3.11	5.14	1.01E-07	5.14E-05
divalent inorganic cation homeostasis (GO:0072507)	466	12	2.4	5	6.08E-06	1.92E-03
ion homeostasis (GO:0050801)	743	19	3.83	4.96	9.46E-09	7.44E-06
regulation of growth (GO:0040008)	679	17	3.5	4.86	8.49E-08	4.61E-05
cellular chemical homeostasis (GO:0055082)	693	17	3.57	4.76	1.13E-07	5.55E-05
cation homeostasis (GO:0055080)	664	16	3.42	4.68	3.52E-07	1.58E-04
inorganic ion homeostasis (GO:0098771)	676	16	3.48	4.59	4.45E-07	1.94E-04

cellular homeostasis (GO:0019725)	842	18	4.34	4.15	3.36E-07	1.56E-04
regulation of cellular response to stress (GO:0080135)	654	12	3.37	3.56	1.53E-04	3.31E-02
chemical homeostasis (GO:0048878)	1050	19	5.41	3.51	1.79E-06	6.40E-04
negative regulation of cell death (GO:0060548)	999	16	5.15	3.11	5.61E-05	1.38E-02
homeostatic process (GO:0042592)	1558	24	8.03	2.99	1.05E-06	4.15E-04
regulation of apoptotic process (GO:0042981)	1510	21	7.78	2.7	2.66E-05	7.34E-03
regulation of programmed cell death (GO:0043067)	1523	21	7.85	2.68	3.01E-05	7.91E-03
regulation of cell death (GO:0010941)	1643	22	8.47	2.6	2.92E-05	7.93E-03
cellular response to stress (GO:0033554)	1582	21	8.15	2.58	5.22E-05	1.30E-02
cellular response to chemical stimulus (GO:0070887)	2674	33	13.78	2.4	1.20E-06	4.59E-04
response to stress (GO:0006950)	3290	40	16.95	2.36	5.30E-08	3.21E-05
response to organic substance (GO:0010033)	2787	31	14.36	2.16	2.13E-05	6.09E-03
negative regulation of biological process (GO:0048519)	5021	53	25.87	2.05	1.31E-08	9.35E-06
response to chemical (GO:0042221)	4172	43	21.5	2	1.93E-06	6.76E-04
regulation of biological quality (GO:0065008)	3814	36	19.65	1.83	1.30E-04	2.88E-02
negative regulation of cellular process (GO:0048523)	4482	42	23.09	1.82	3.82E-05	9.87E-03
response to stimulus (GO:0050896)	7985	61	41.14	1.48	8.95E-05	2.10E-02

Column 1: PANTHER GO-term category. Column 2: Number of reference genes in each category. Column 3: GO term distribution of 100 most up-regulated genes in response to gentamicin-treated flat renal organoids. Column 4: Number of genes expected based on chance. Column 5: Fold-enrichment of the up-regulated genes in each GO term category. Column 6: Significance of gene enrichment compared to expected (*p* value). Column 7: Significance after applying False Discovery Rate (FDR) adjustment.

Table S3. - Significant fold-changes of *HMOX1* biomarker gene expression in flat and 3D *in vitro* kidney organoids after 24h drug treatment, Related to Figure 6 and Figure S5.

**Drug**	**Organoid Type**	**Concentration (low → high)**			
Dexamethasone	Flat	ns	ns	ns	ns
	3D	ns	ns	ns	ns
Ketoprofen	Flat	ns	ns	ns	ns
	3D	ns	ns	ns	ns
Puromycin	Flat	ns	*	*	**
	3D	ns	ns	*	**
Cisplatin	Flat	ns	*	*	N/A
	3D	ns	**	ns	N/A
Gentamicin	Flat	*	*	ns	ns
	3D	ns	ns	ns	ns
Cidofovir	Flat	ns	ns	ns	ns
	3D	ns	ns	ns	ns
Ifosfamide	Flat	ns	ns	ns	ns
	3D	ns	ns	ns	ns

Significance determined by repeated measure one-way ANOVA on $2^{-\Delta\Delta CT}$ values compared to the vehicle control, ns: non-significant, * < 0.05, ** < 0.01, increase indicated in blue, decrease indicated in red.

Table S4. Summary of gRNA and primer sequences for RT-PCR, and TaqMan assay numbers, Related to Figure 3A.

**Description/Name**	**Sequence**
HMOX1 gRNA1 for CRISPR targeting	CACCGGCTTTATGCCATGTGAATGC
HMOX1 gRNA2 for CRISPR targeting	CACCGGCCAGCATGCCTGCATTCACA
Human *OAT1 (SLC22A6)* forward primer	AGTCCTTGTACATGGTGGGG
Human *OAT1 (SLC22A6*) reverse primer	CATGCAGTTGAGGGAGATGC
Human *OCT2 (SLC22A2)* forward primer	CCGTAAGCTCTGCCTCCTAA
Human *OCT2 (SLC22A2)* reverse primer	TGTTCTCCGATATCTCCGCC
Human *Megalin (LRP2)* forward primer	AACGAGACGCACAGTAGTTCA
Human *Megalin (LRP2)* reverse primer	GTGAAGCAGCCCAATCGATG
Human *Cubulin (CUBN)* forward primer	TCGTCTCCTCAGGAAACAGC
Human *Cubulin (CUBN)* reverse primer	TGGAGGTTGCGCTTGAATGA
HMOX1 Targeting verification primer 1	AGGGATGGGACTGAACTTGA
β-actin (ACTB) forward primer	CTGGGACGACATGGAGAAAA
β-actin (ACTB) reverse primer	AAGGAAGGCTGGAAGAGTGC
HMOX1 Targeting verification primer 2	ACCTTGAAGCGCATGAACTC
HMOX1 Targeting verification primer 3	ACAACGAGGACTACACCATC
HMOX1 Targeting verification primer 4	AATATTCGCCCACCAGCTAC
Hprt1 TaqMan Reference ID	Mm00446968_m1
HMOX1 (HO1) Taqman Reference ID	Hs01110250_m1

Table S5. The 100 most up-regulated genes classified by overlap in various gene categories that they belong to using GO classification, Related to Figure 2.

Names	Total	Elements
Binding MF, Biolog Regul BP, Catal Act MF, Cellul Proc BP, Metab Proc BP, Resp to Stim BP	2	TRIB3, HMOX1
Binding MF, Catal Act MF, Cellul Proc BP, Metab Proc BP, Resp to Stim BP	3	HSPA6, HSPA1B, HSPA1A
Binding MF, Catal Act MF, Cellul Proc BP, Metab Proc BP	3	ASNS, FKBP4, TSPYL2
Binding MF, Cellul Proc BP, Metab Proc BP, Resp to Stim BP	2	INHBE, MSTN
Binding MF, Biolog Regul BP, Cellul Proc BP, Metab Proc BP	1	HIST1H2AG
Binding MF, Biolog Regul BP, Cellul Proc BP, Resp to Stim BP	2	LRG1, EGF
Catal Act MF, Cellul Proc BP, Metab Proc BP, Resp to Stim BP	1	PPP1R15A
Biol Regul BP, Catal Act MF, Cellul Proc BP, Metab Proc BP	3	DIO3, GCLM, CES1
Binding MF, Cellul Proc BP, Metab Proc BP	6	SFBQ, PSPC1, GCM1, DNAJB1, ATF5, JDP2
Binding MF, Cellul Proc BP, Resp to Stim BP	1	LTA
Binding MF, Biol Regul BP, Cellul Proc BP	2	FTH1, FTL
Binding MF, Biolog Regul BP, Metab Proc BP	1	ID2
Catal Act MF, Cellul Proc BP, Metab Proc BP	6	UAP1L1, WARS, BAAT, HTRA3, B3GALNT2, ABCC3
Biolog Regul BP, Cellul Proc BP, Resp to Stim BP	3	CASS4, SLC30A2, GABRR2
Binding MF, Cellul Proc BP	3	CHORDC1, TUBB3, AVIL
Binding MF, Metab Proc BP	1	BRPF3
Catal Act MF, Cellul Proc BP	2	ME1, NEURL3
Catal Act MF, Metab Proc BP	2	CHKA, ASPRV1
Cellul Proc BP, Metab Proc BP	2	SLC1A4, ZNF425
Binding MF	4	ZFAND2A, CNN1, SQSTM1, MRPL18

Cellul Proc BP	8	SLC38A10, SLC7A11, GATA2, MC1R, RRAD, SCFD2, MYH15, RASL11A
Metab Proc BP	1	ANXA1
